# Supplementary figures and images for: AUM302, a novel triple kinase PIM/PI3K/mTOR inhibitor, is a potent in vitro pancreatic cancer growth inhibitor
Source: PLoS One. 2023 Nov 9;18(11):e0294065. doi: 10.1371/journal.pone.0294065 (PMC10635512; doi:10.1371/journal.pone.0294065)

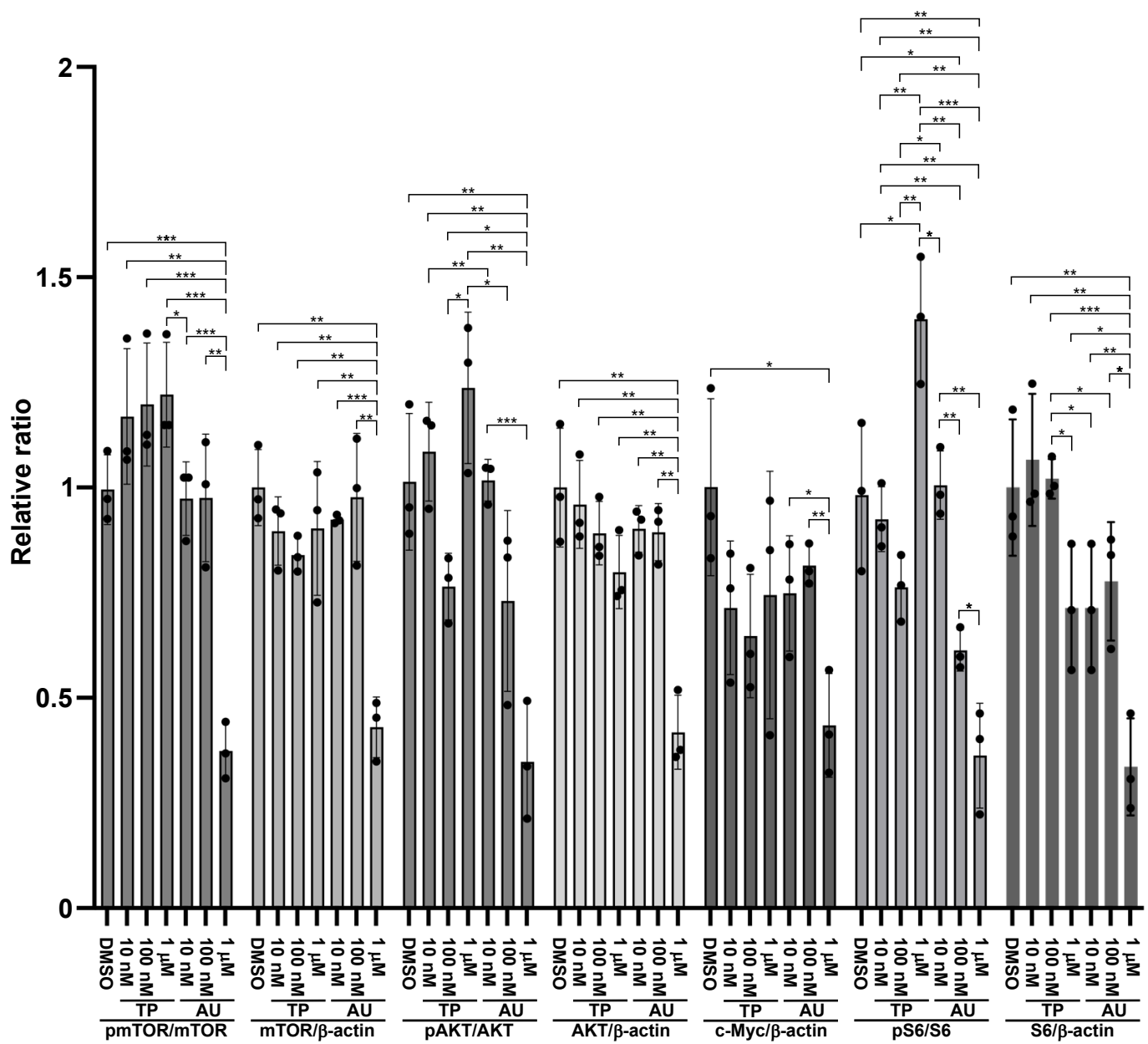

Supplementary Figure 1.

Supplement: S1 Fig — Each experiment was performed in triplicate and the results are shown as mean ±SD (N = 3). Densitometry analysis was performed using FIJI software [61]. Statistical analysis was performed using the Student’s test followed by an analysis of the normal distribution (Tukey’s test). *p<0.05; **p<0.01; ***p<0.001. (PDF) [file pone.0294065.s001.pdf]

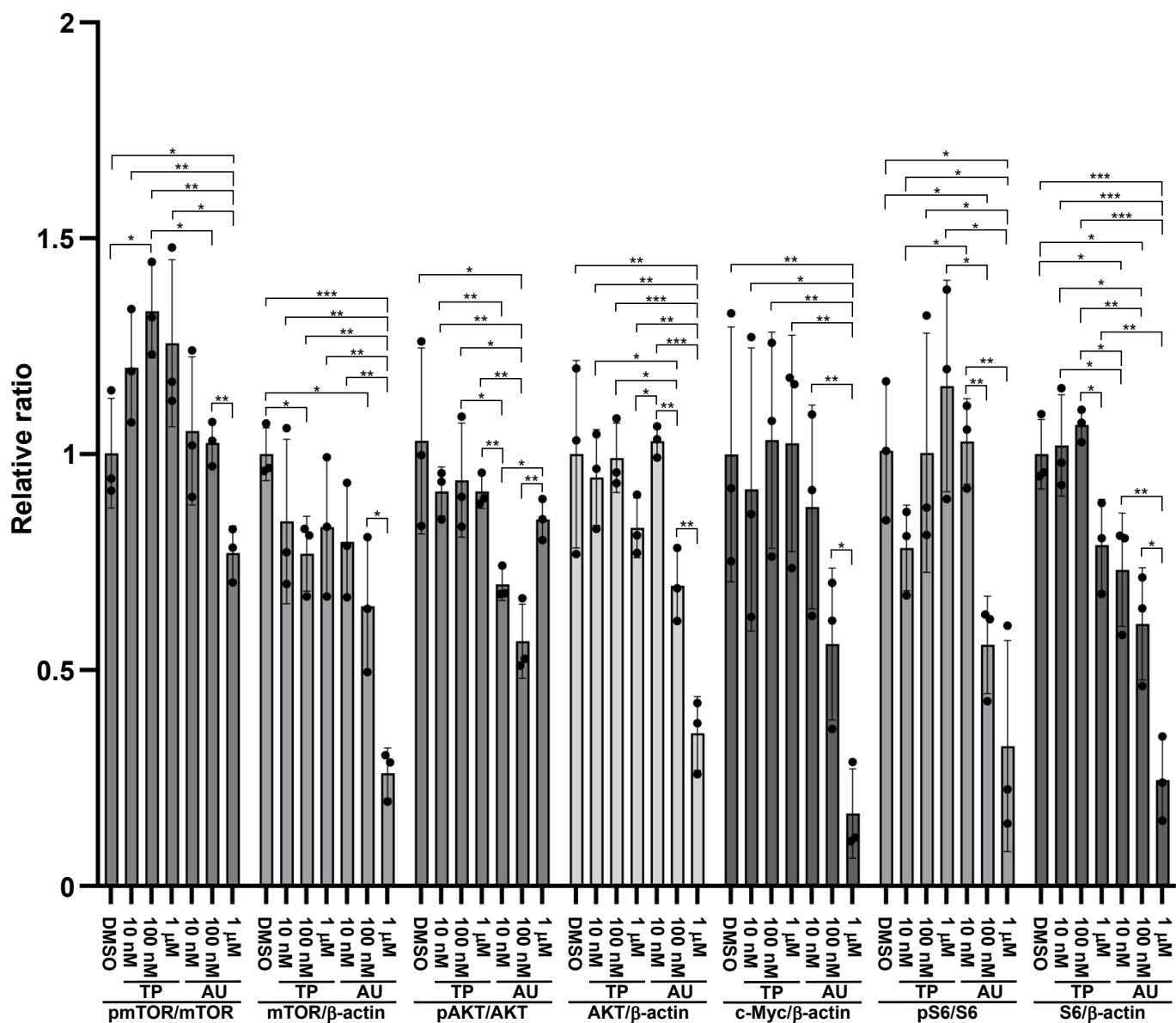

Supplementary Figure 2.

Supplement: S2 Fig — Each experiment was performed in triplicate and the results are shown as mean ±SD (N = 3). Densitometry analysis was performed using FIJI software [61]. Statistical analysis was performed using the Student’s test followed by an analysis of the normal distribution (Tukey’s test). *p<0.05; **p<0.01; ***p<0.001. (PDF) [file pone.0294065.s002.pdf]

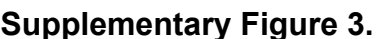

Supplement: S3 Fig — Each experiment was performed in triplicate and the results are shown as mean ±SD (N = 3). Densitometry analysis was performed using FIJI software [61]. Statistical analysis was performed using the Student’s test followed by an analysis of the normal distribution (Tukey’s test). *p<0.05; **p<0.01; ***p<0.001. (PDF) [file pone.0294065.s003.pdf]
