# Supplementary material for: AUM302, a novel triple kinase PIM/PI3K/mTOR inhibitor, is a potent in vitro pancreatic cancer growth inhibitor
Source: PLoS One. 2023 Nov 9;18(11):e0294065. doi: 10.1371/journal.pone.0294065 (PMC10635512; doi:10.1371/journal.pone.0294065)

BxPC-3 24h phospho-mTOR

Method: Azure
Figure 6A (boxed panel)

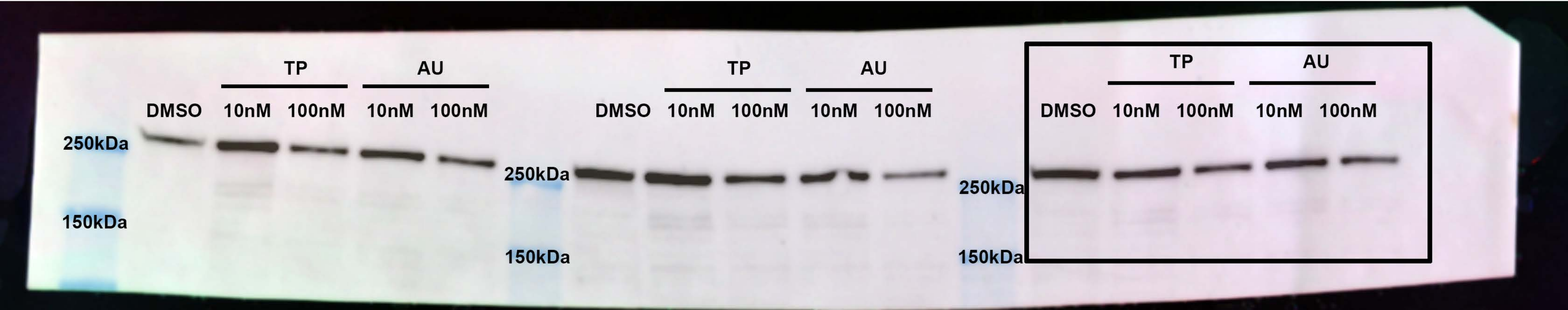

Method: Azure
Figure 6A (boxed panel)

BxPC-3 24h mTOR

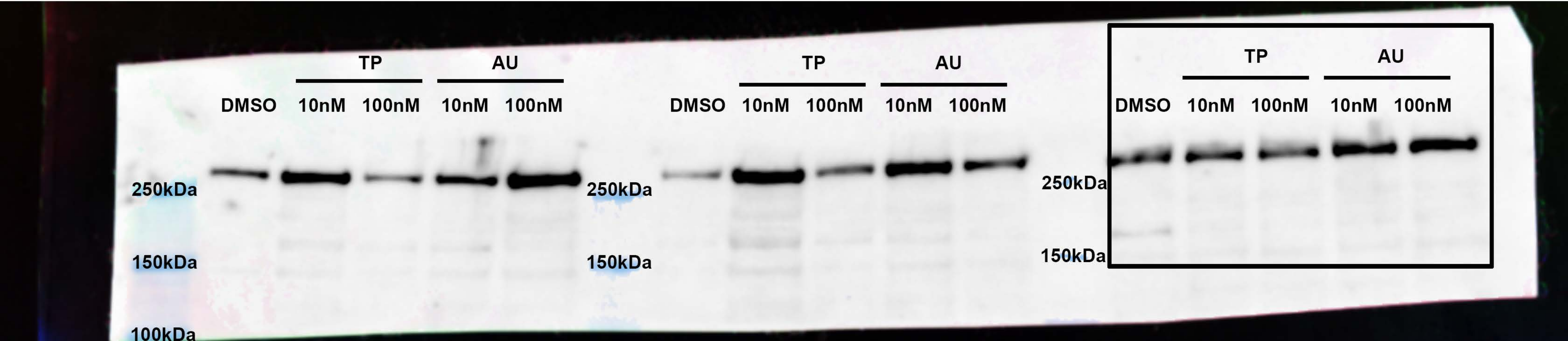

BxPC-3 24h phospho-AKT

Method: Azure
Figure 6A (boxed panel)

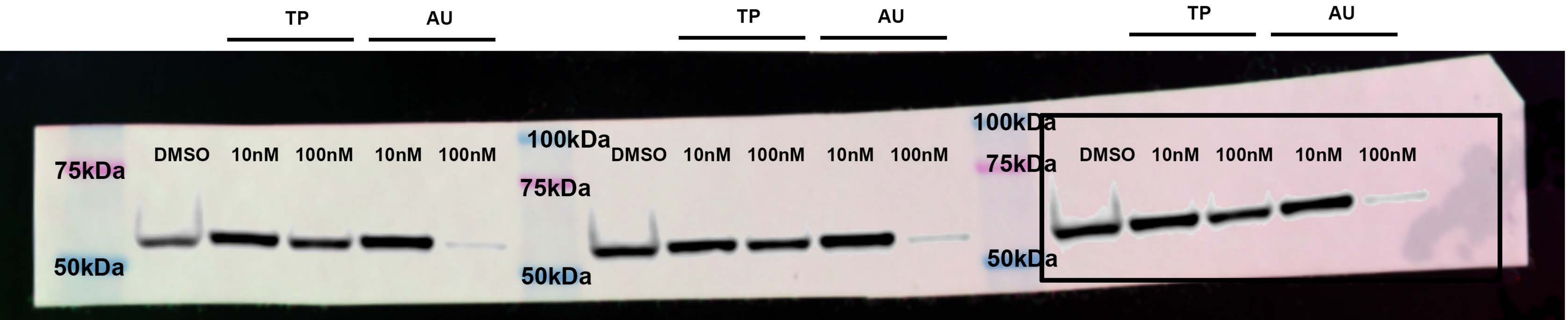

BxPC-3 24h AKT

Method: Azure
Figure 6A (boxed panel)

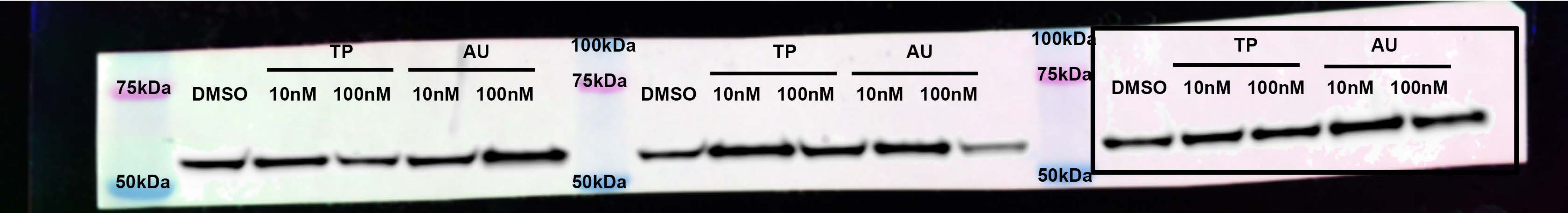

BxPC-3 24h c-Myc

Method: Azure
Figure 6A (boxed panel)

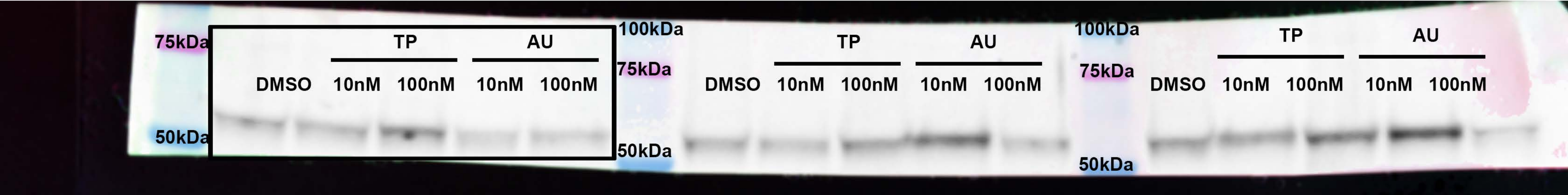

BxPC-3 24h phospho-S6

Method: Azure
Figure 6A (boxed panel)

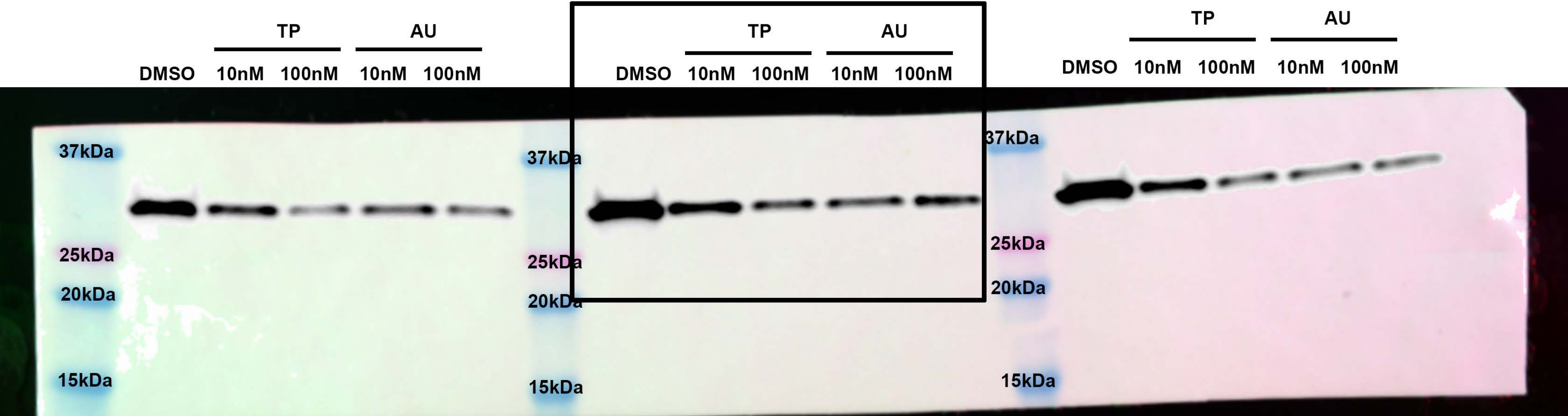

BxPC-3 24h S6

Method: Azure
Figure 6A (boxed panel)

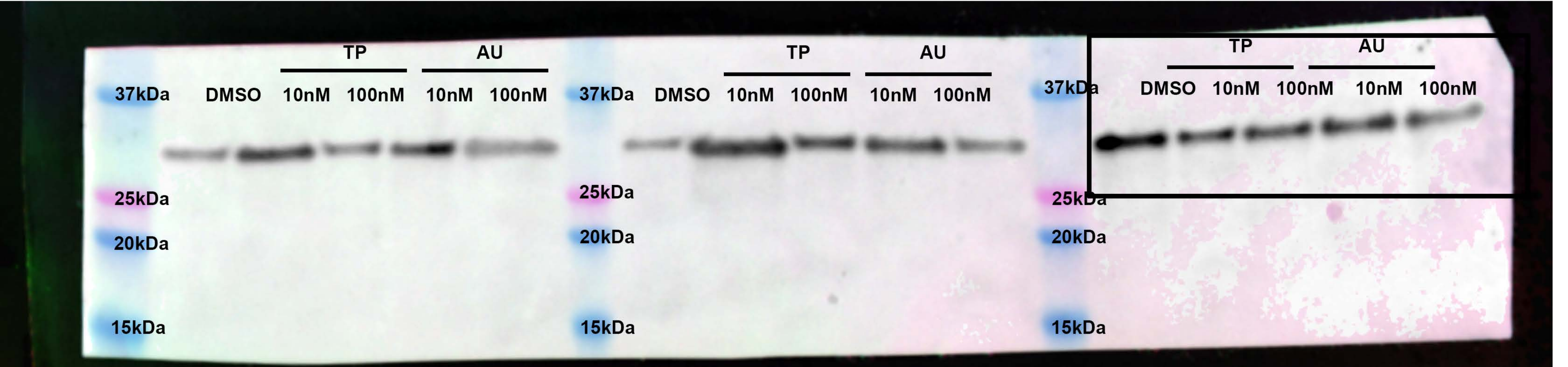

BxPC-3 24h Actin

Method: Azure
Figure 6A (boxed panel)

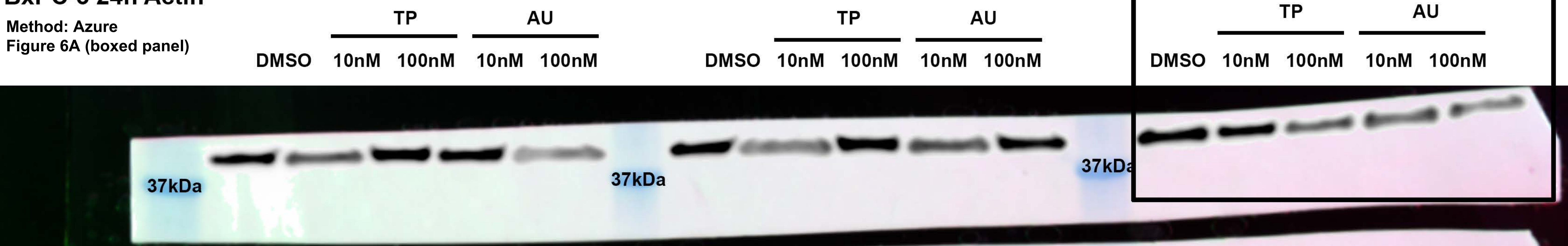

Capan-2 24h phospho-mTOR

Method: Azure
Figure 6B (boxed panel)

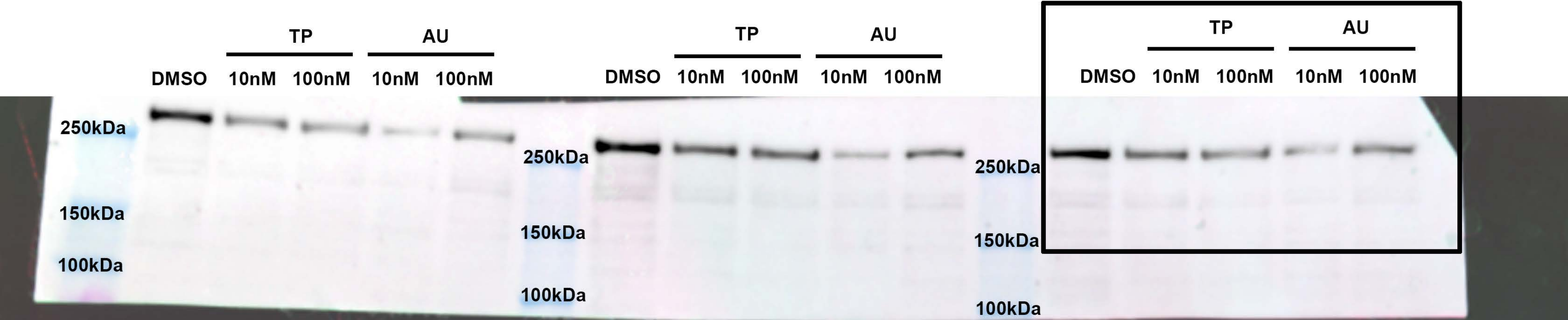

Capan-2 24h mTOR

Method: Azure
Figure 6B (boxed panel)

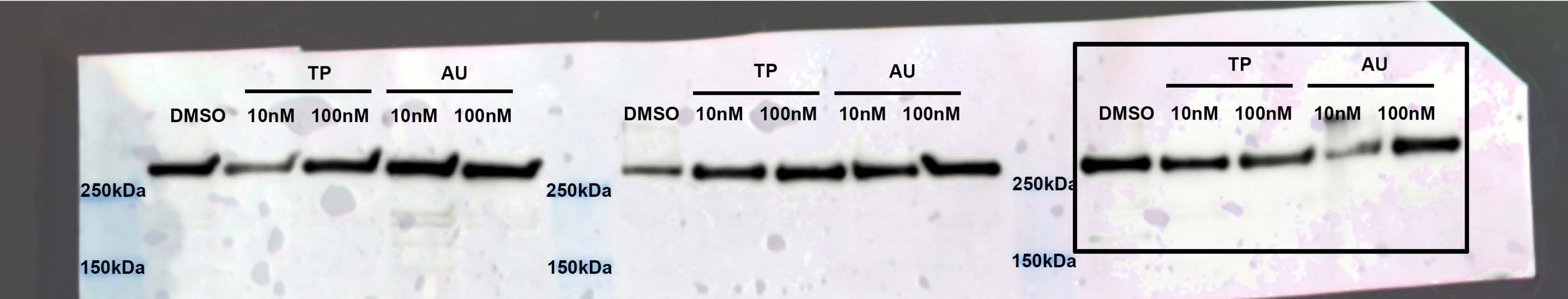

Capan-2 24h phospho-AKT

Method: Azure
Figure 6B (boxed panel)

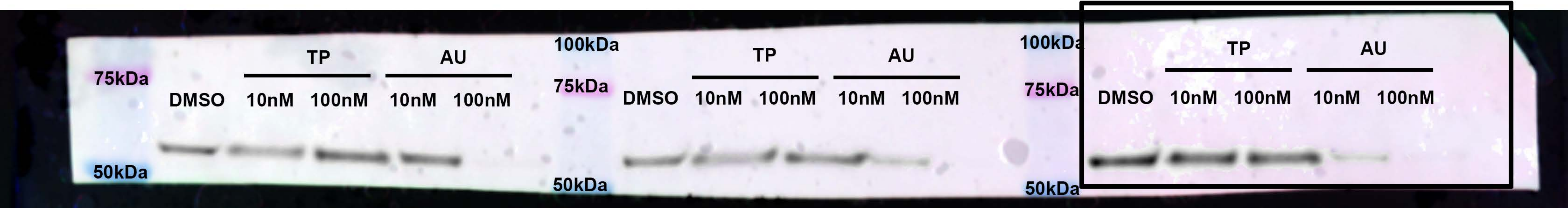

Capan-2 24h AKT

Method: Azure
Figure 6B (boxed panel)

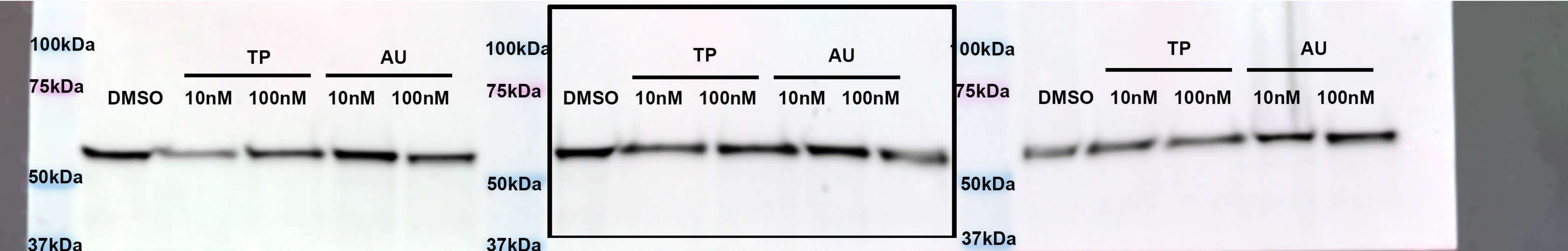

Capan-2 24h c-Myc

Method: Azure
Figure 6B (boxed panel)

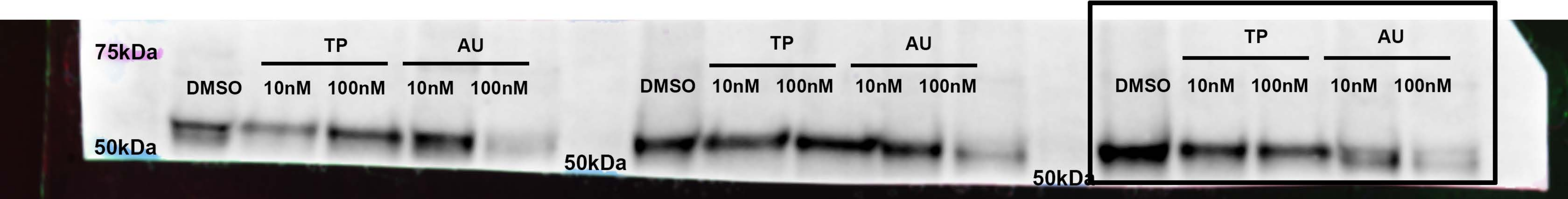

Capan-2 24h phospho-S6

Method: Azure
Figure 6B (boxed panel)

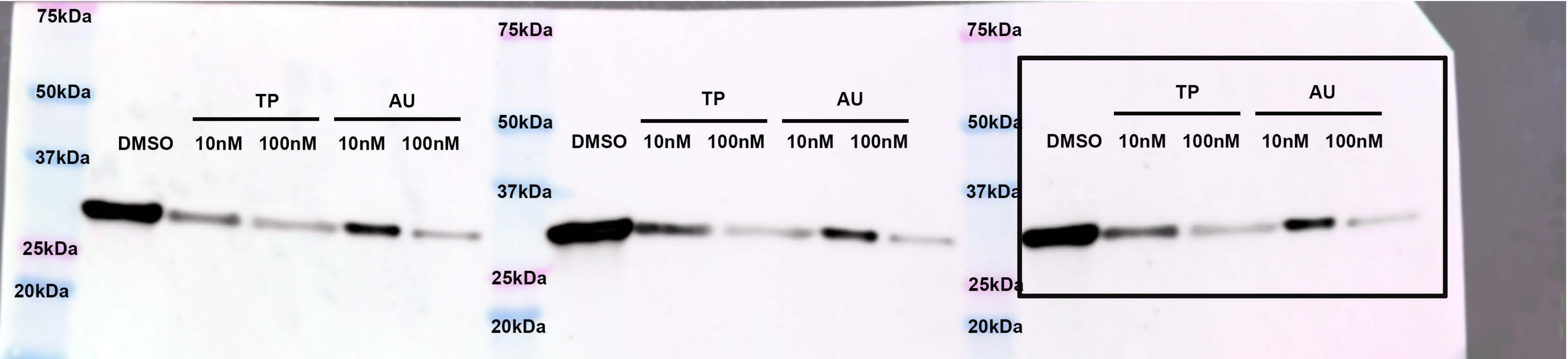

Capan-2 24h S6

Method: Azure
Figure 6B (boxed panel)

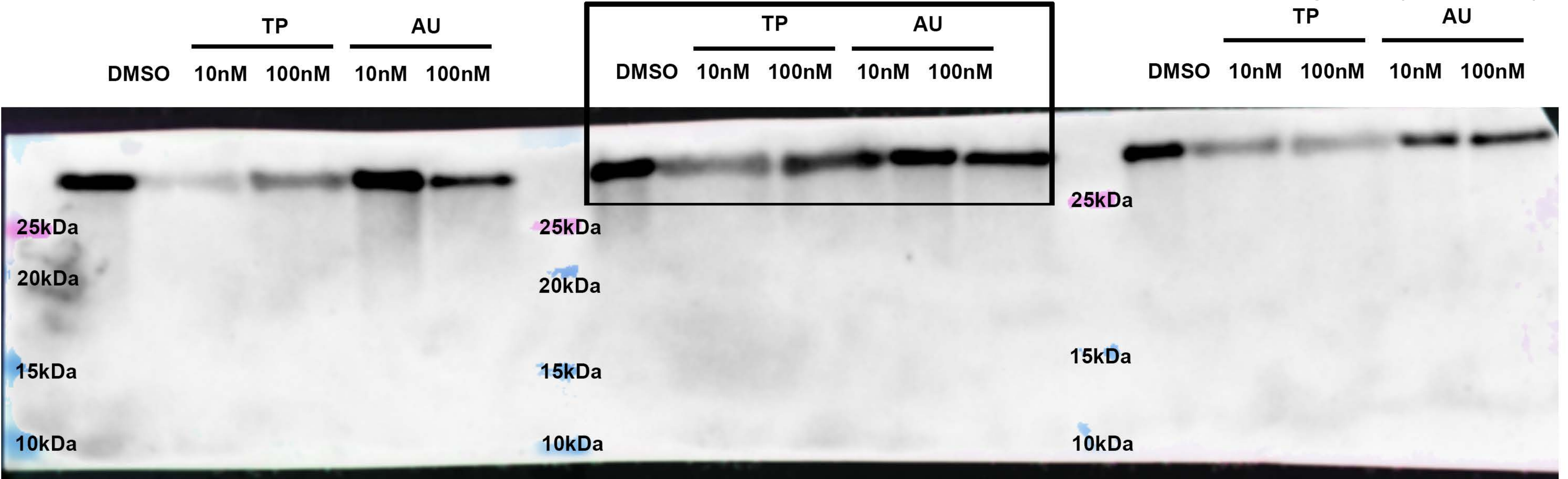

Capan-2 24h Actin

Method: Azure
Figure 6B (boxed panel)

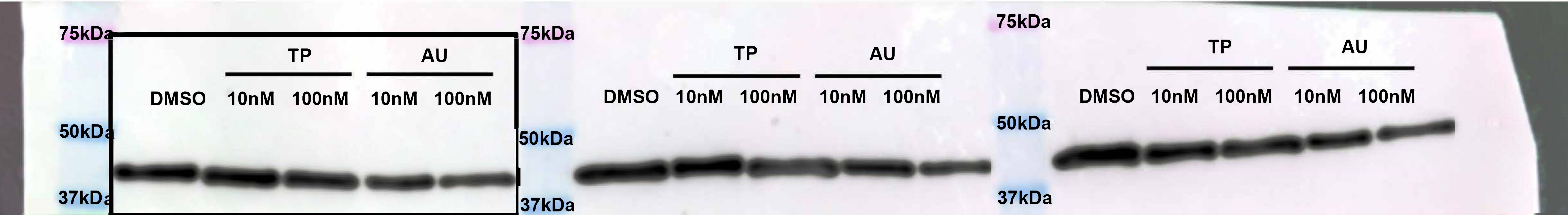

MIA PaCA-2 24h phospho-mTOR

Method: Azure
Figure 6C (boxed panel)

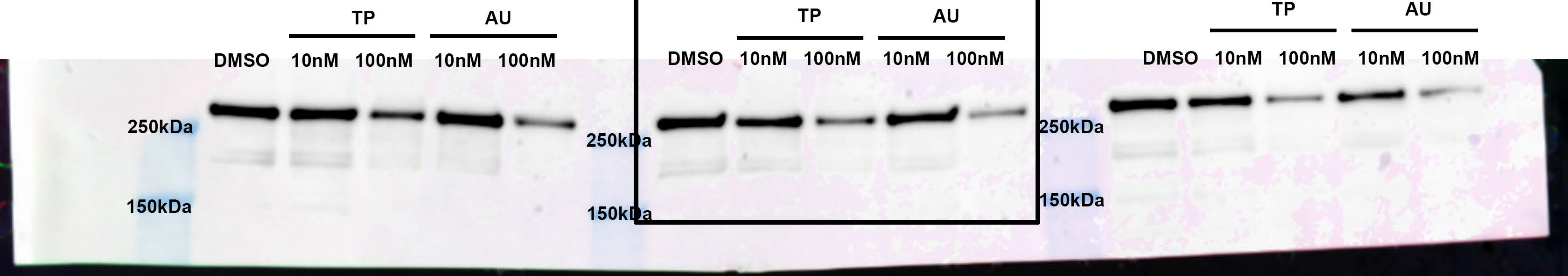

Method: Azure
Figure 6C (boxed panel)

MIA PaCA-2 24h mTOR

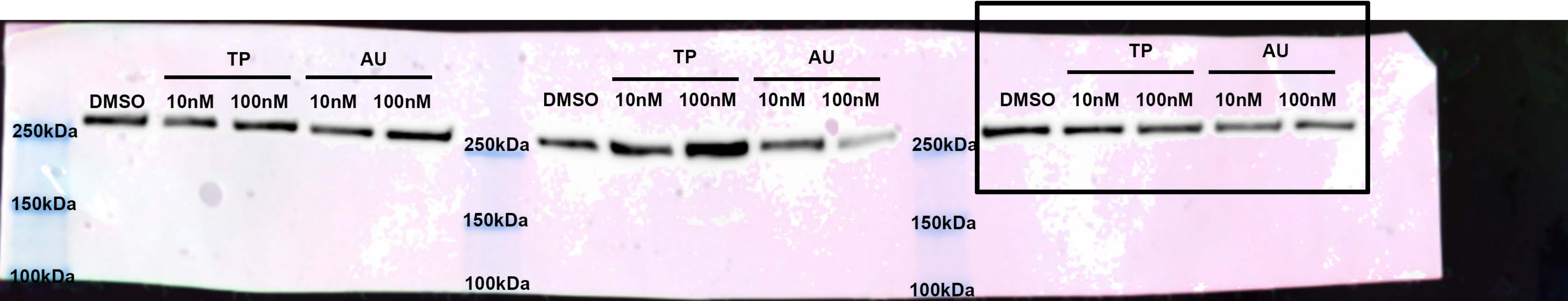

MIA PaCA-2 24h phospho-AKT

Method: Azure
Figure 6C (boxed panel)

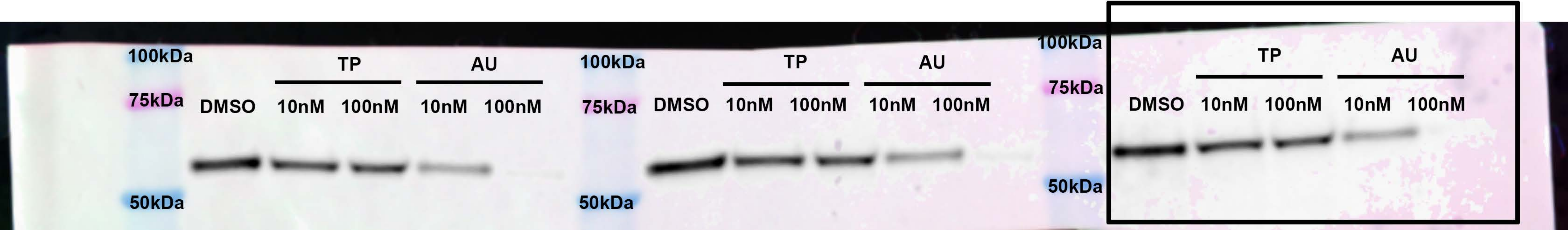

MIA PaCA-2 24h AKT

Method: Azure
Figure 6C (boxed panel)

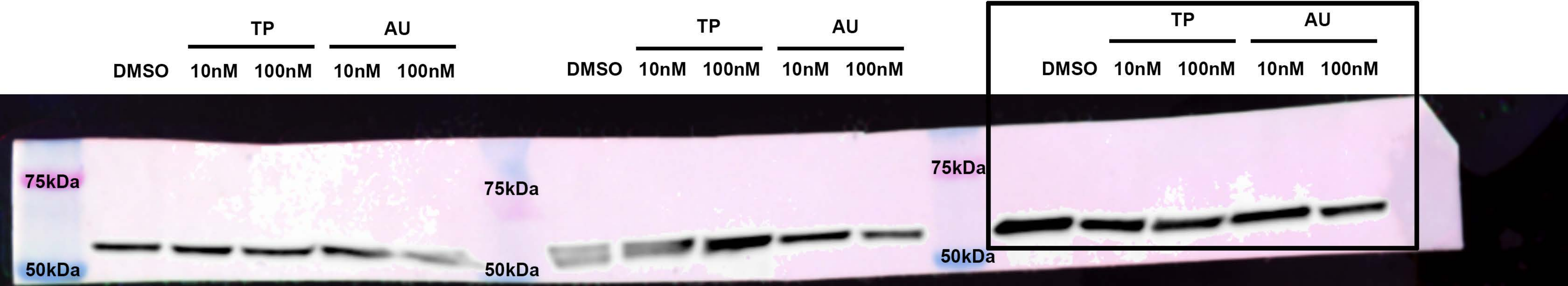

MIA PaCA-2 24h c-Myc

Method: Azure
Figure 6C (boxed panel)

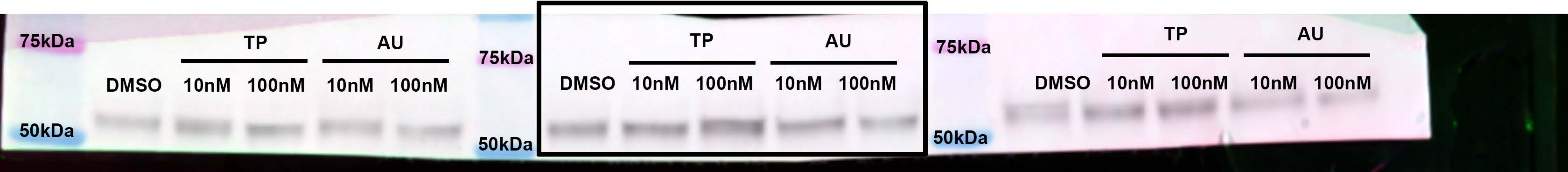

MIA PaCA-2 24h phospho-S6

Method: Azure
Figure 6C (boxed panel)

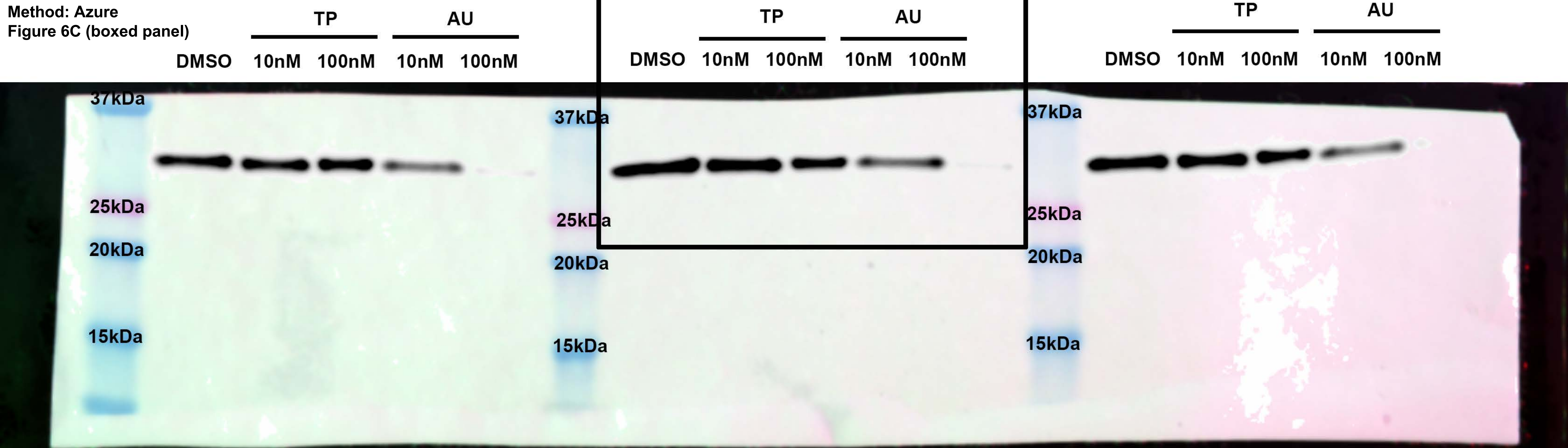

MIA PaCA-2 24h S6

Method: Azure
Figure 6C (boxed panel)

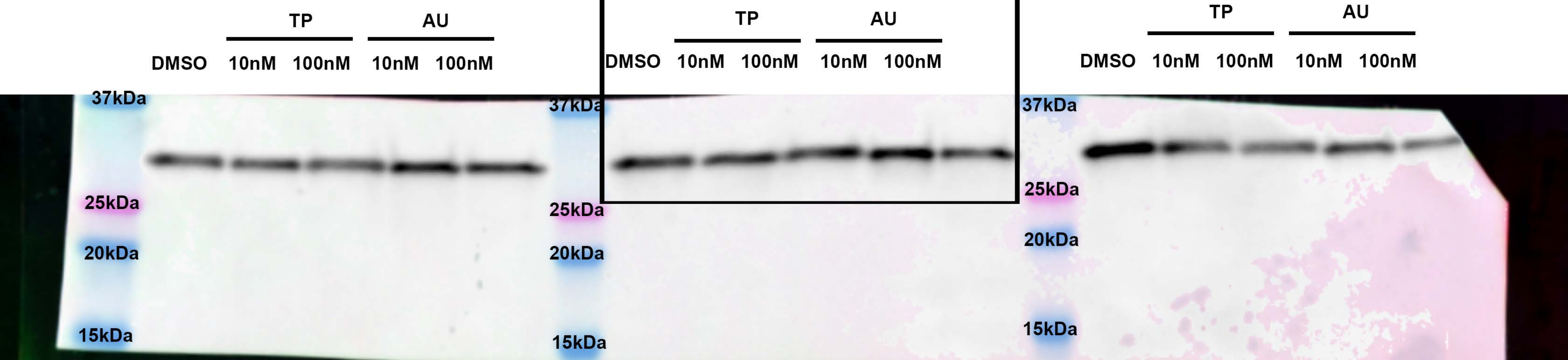

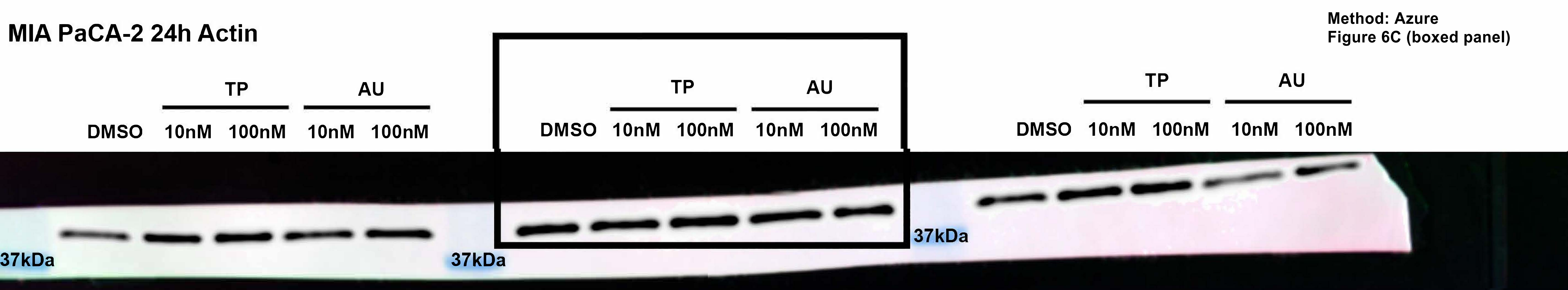
MIA PaCA-2 24h Actin
Method: Azure
Figure 6C (boxed panel)
TP
AU
DMSO
10nM
100nM
10nM
100nM
TP
AU
DMSO
10nM
100nM
10nM
100nM
TP
AU
DMSO
10nM
100nM
10nM
100nM
37kDa
37kDa
37kDa

PANC-1 24h phospho-mTOR

Method: Azure
Figure 6D (boxed panel)

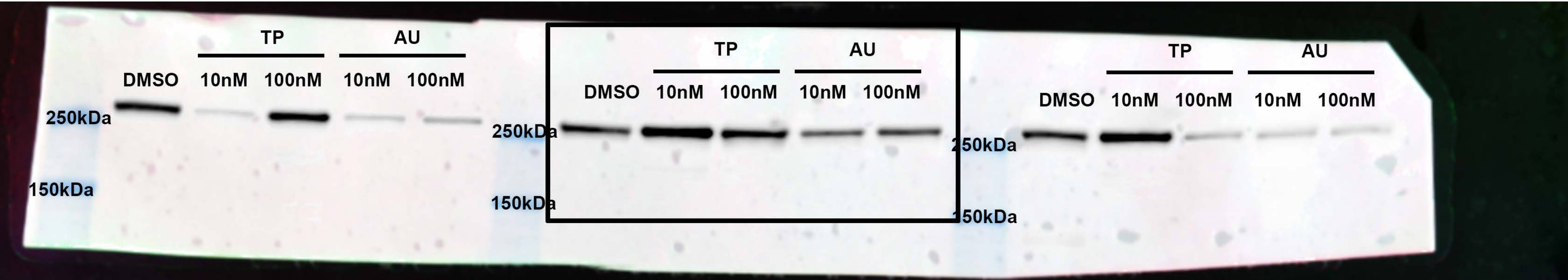

PANC-1 24h mTOR

Method: Azure
Figure 6D (boxed panel)

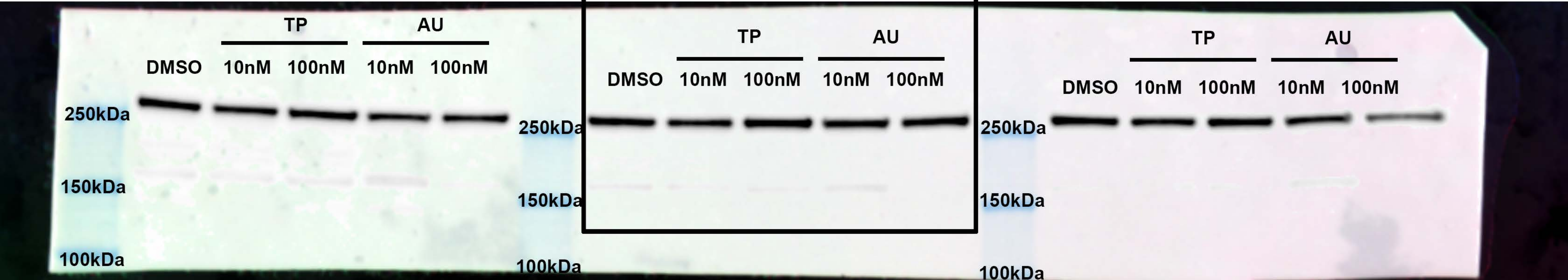

PANC-1 24h phospho-AKT

Method: Azure
Figure 6D (boxed panel)

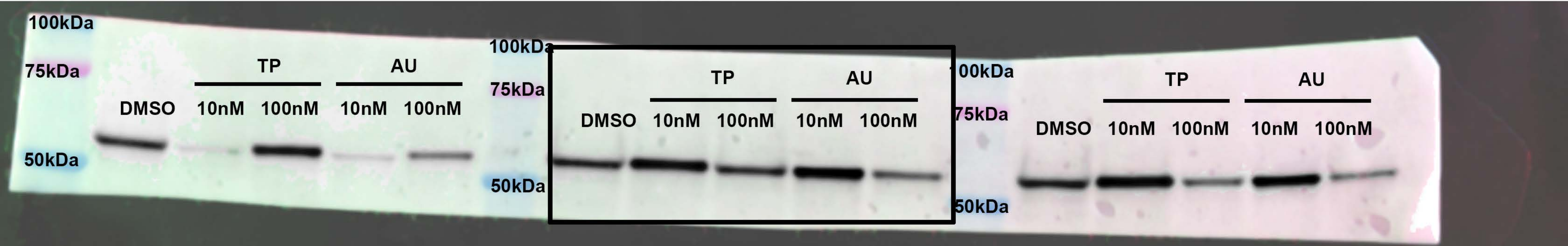

PANC-1 24h AKT

Method: Azure
Figure 6D (boxed panel)

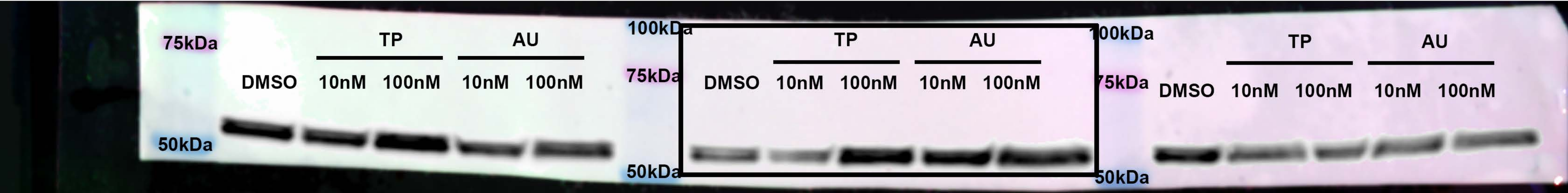

PANC-1 24h c-Myc

Method: Azure
Figure 6D (boxed panel)

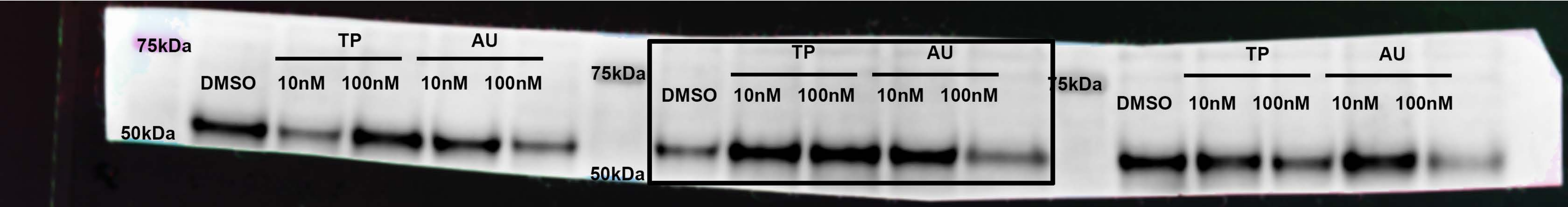

PANC-1 24h phospho-S6

Method: Azure
Figure 6D (boxed panel)

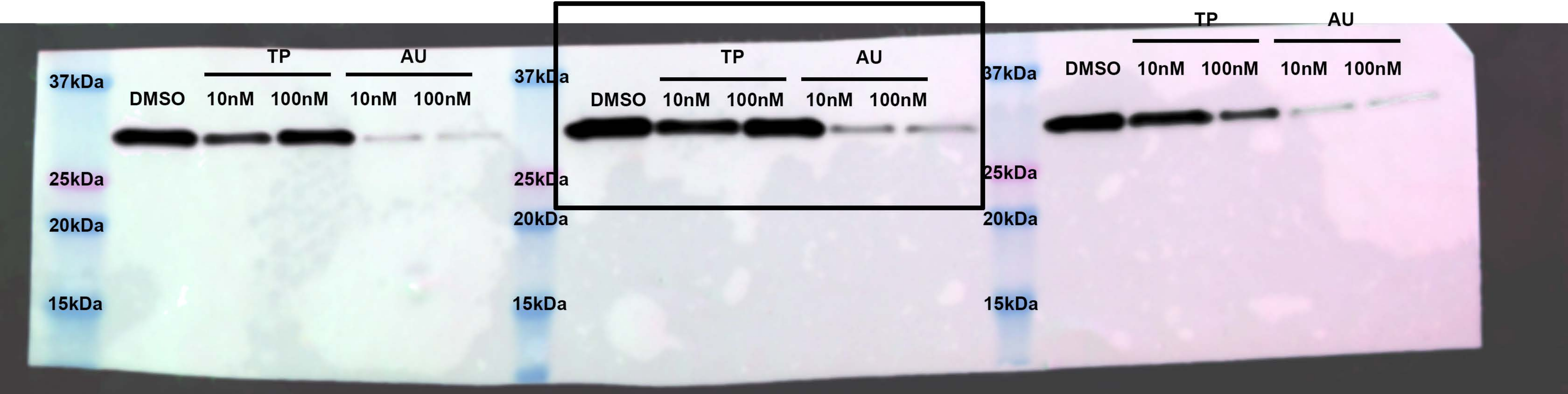

PANC-1 24h S6

Method: Azure
Figure 6D (boxed panel)

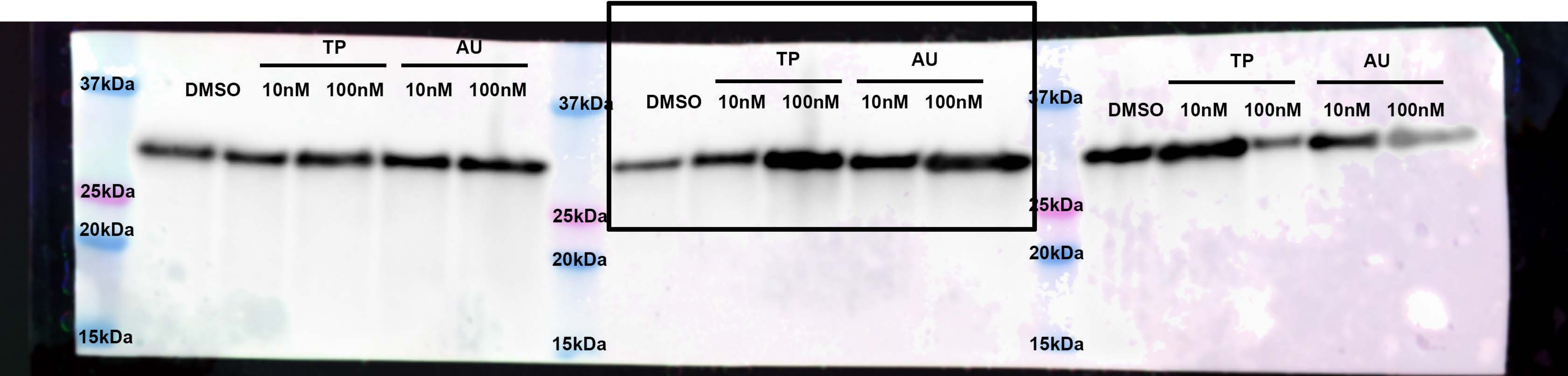

PANC-1 24h Actin

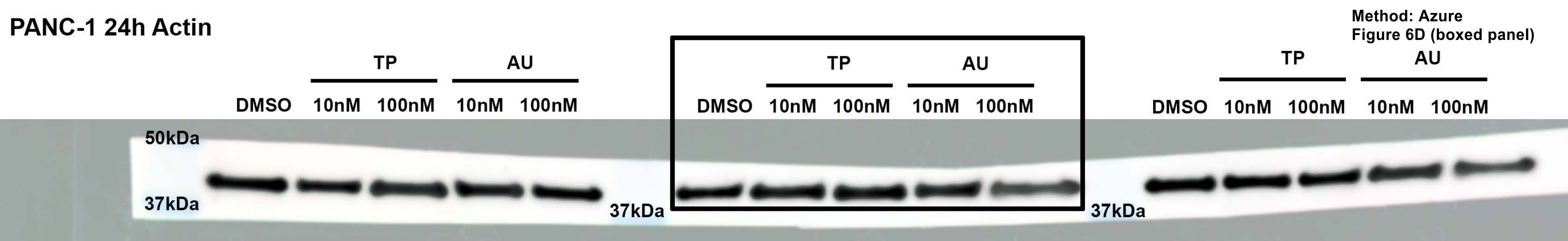

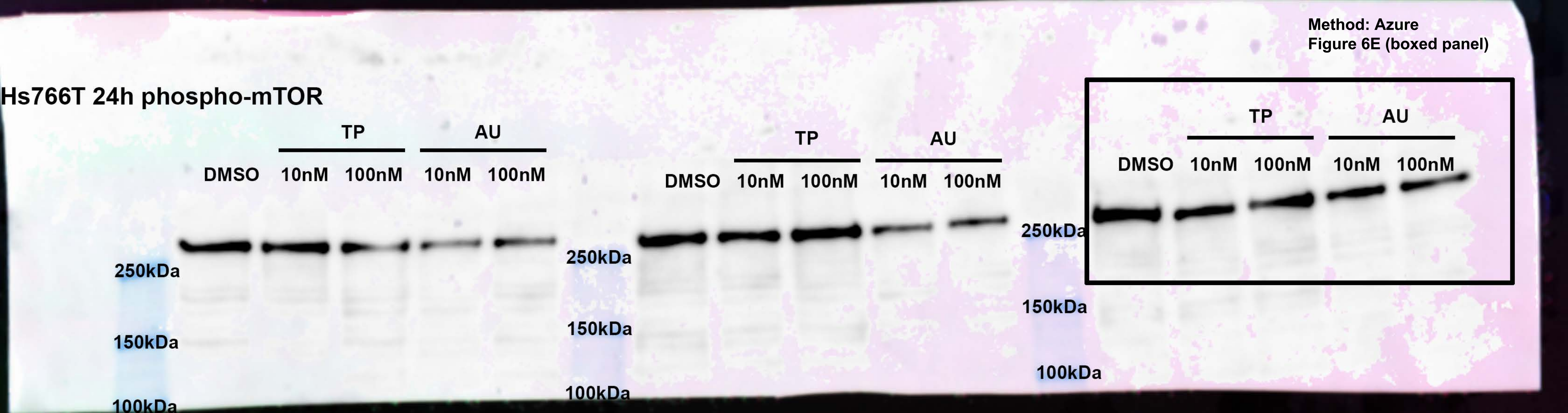
Method: Azure
Figure 6E (boxed panel)
Hs766T 24h phospho-mTOR
TP
AU
DMSO
10nM
100nM
10nM
100nM
250kDa
150kDa
100kDa
TP
AU
DMSO
10nM
100nM
10nM
100nM
250kDa
150kDa
100kDa
TP
AU
DMSO
10nM
100nM
10nM
100nM
250kDa
150kDa
100kDa

Method: Azure
Figure 6E (boxed panel)

Hs766T 24h mTOR

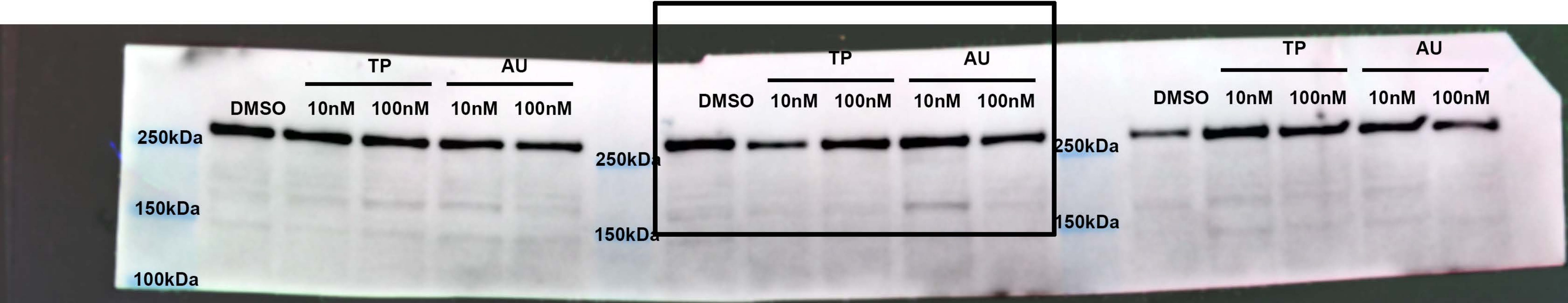

Hs766T 24h phospho-AKT

Method: Azure
Figure 6E (boxed panel)

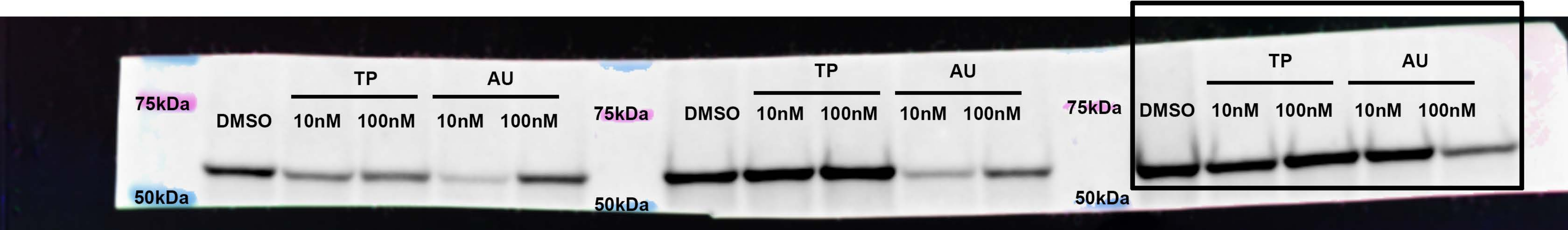

Hs766T 24h AKT

Method: Azure
Figure 6E (boxed panel)

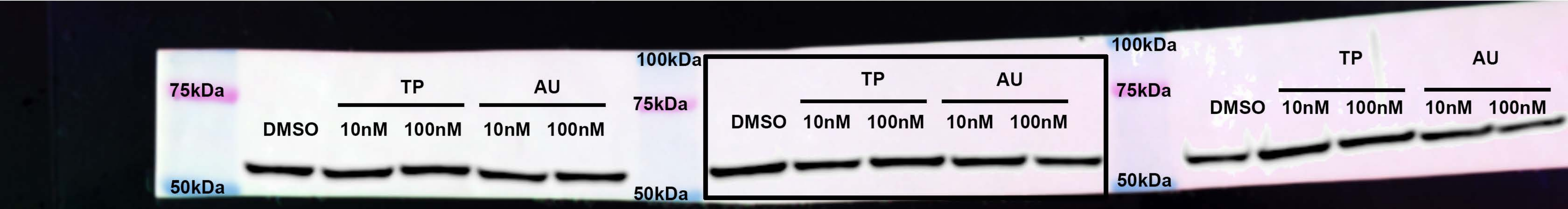

Hs766T 24h c-Myc

Method: Azure
Figure 6E (boxed panel)

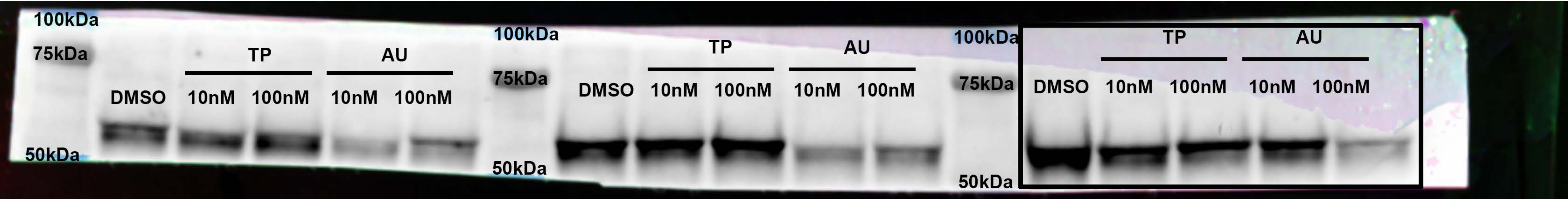

Hs766T 24h phospho-S6

Method: Azure
Figure 6E (boxed panel)

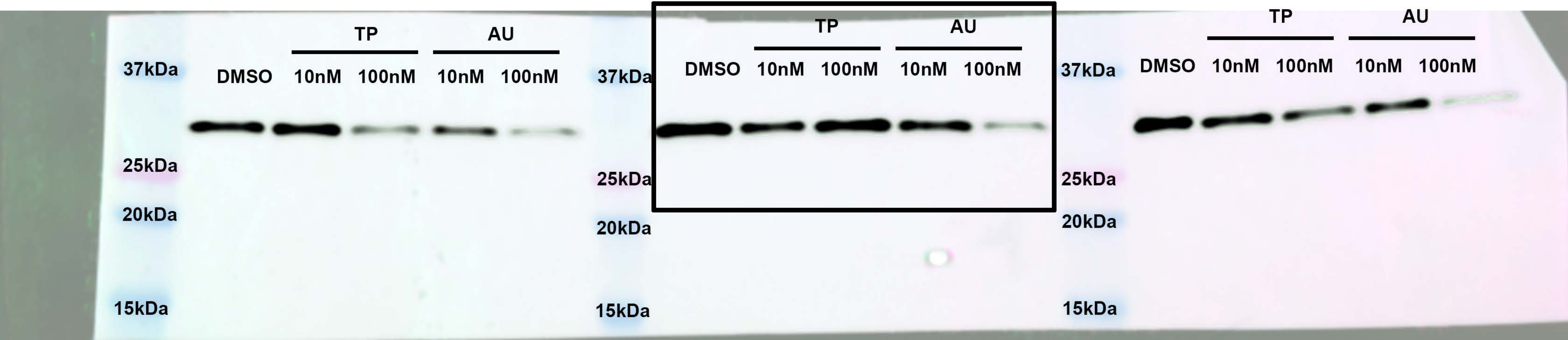

Hs766T 24h S6

Method: Azure
Figure 6E (boxed panel)

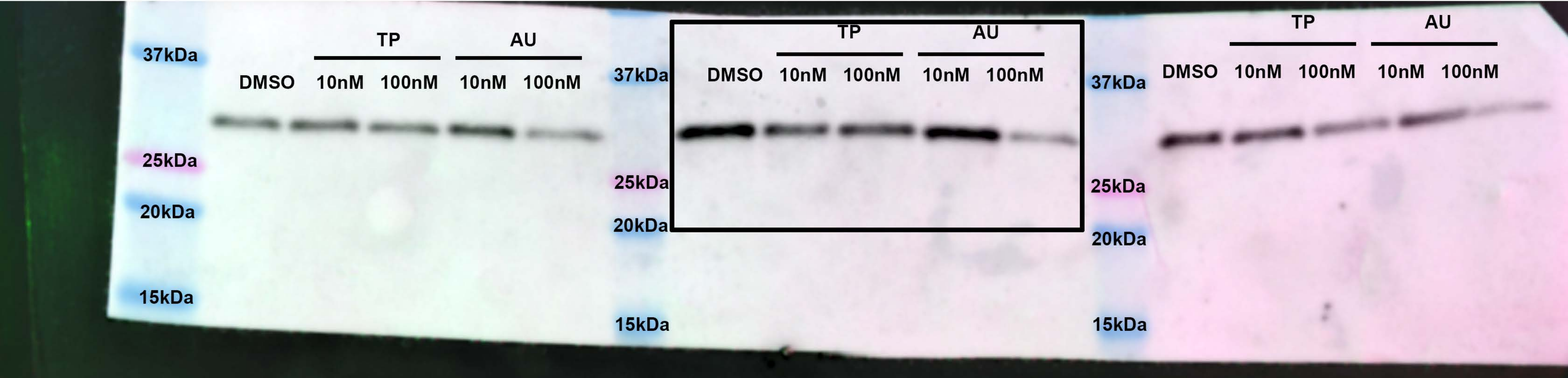

Method: Azure
Figure 6E (boxed panel)

Hs766T 24h Actin

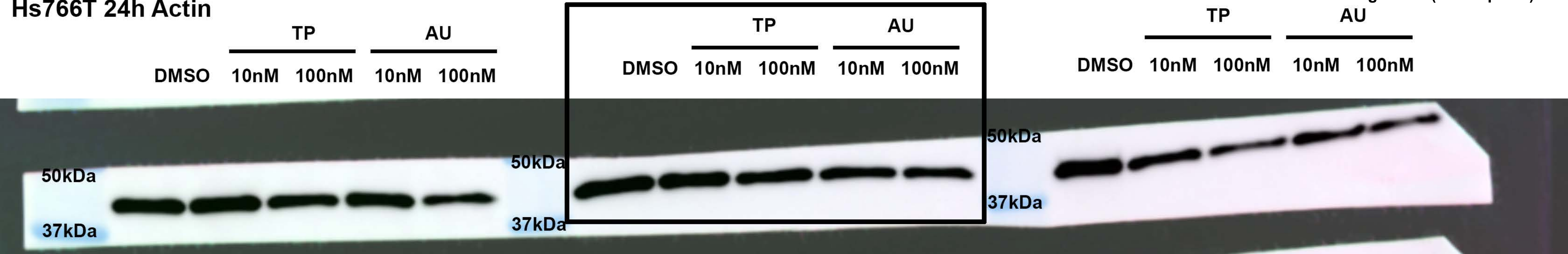

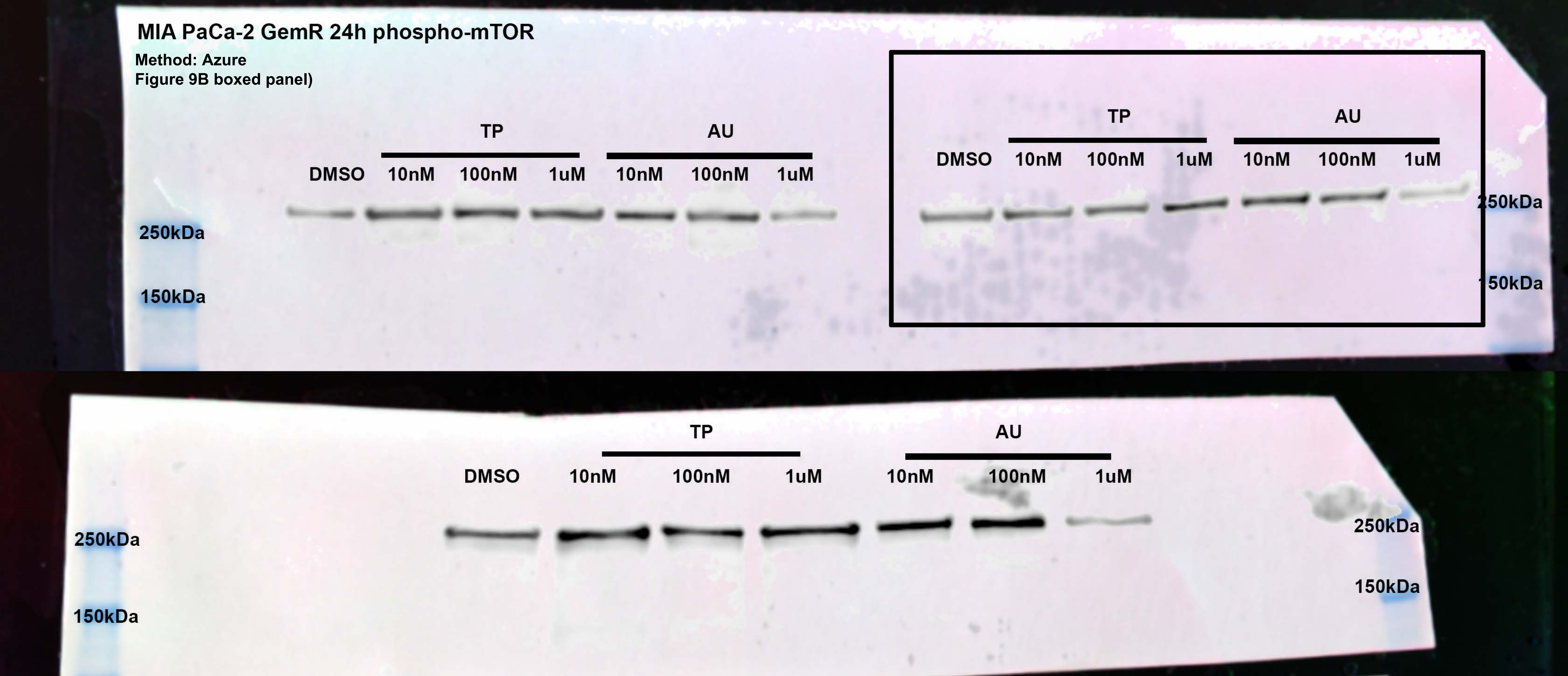
MIA PaCa-2 GemR 24h phospho-mTOR
Method: Azure
Figure 9B boxed panel)
TP
AU
DMSO
10nM
100nM
1uM
10nM
100nM
1uM
250kDa
150kDa
TP
AU
DMSO
10nM
100nM
1uM
10nM
100nM
1uM
250kDa
150kDa
TP
AU
DMSO
10nM
100nM
1uM
10nM
100nM
1uM
250kDa
150kDa
250kDa
150kDa

MIA PaCa-2 GemR 24h mTOR

Method: Azure
Figure 9B boxed panel)

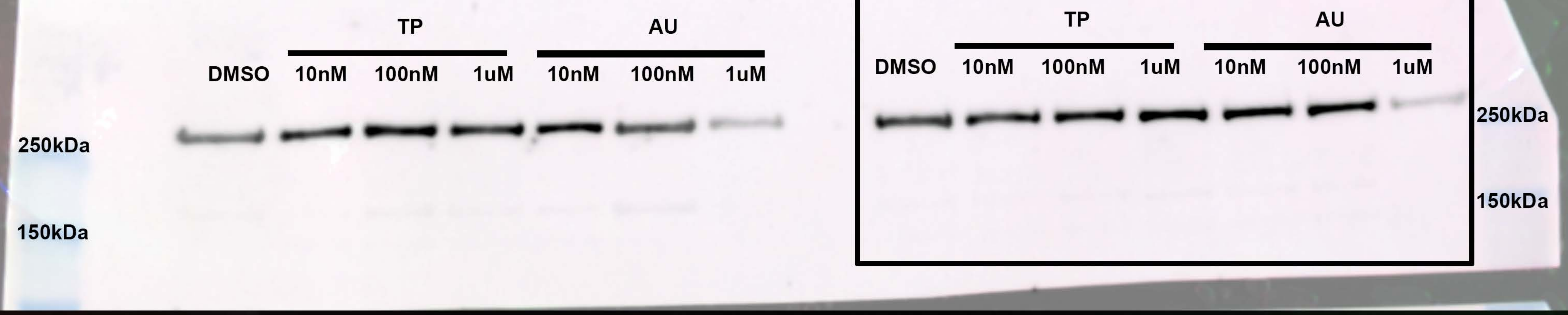

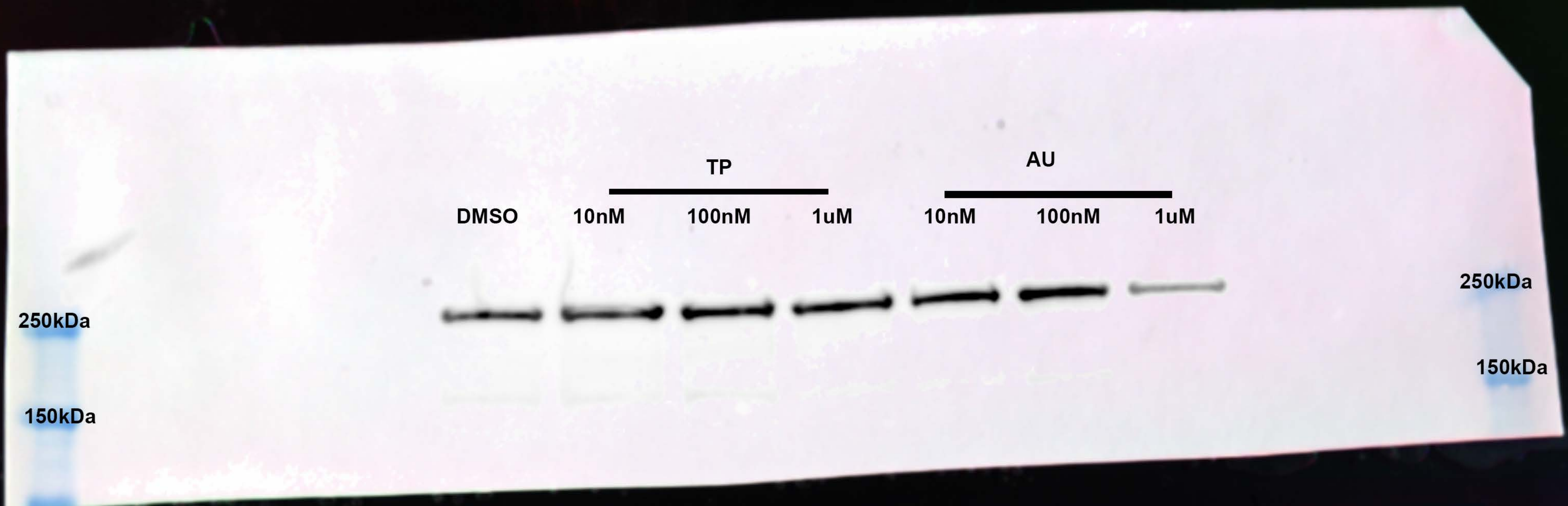

MIA PaCa-2 GemR phospho-AKT

Method: Azure
Figure 9B boxed panel)

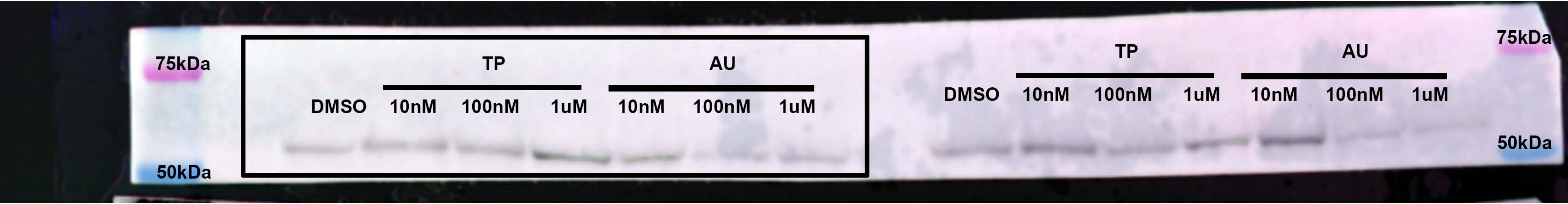

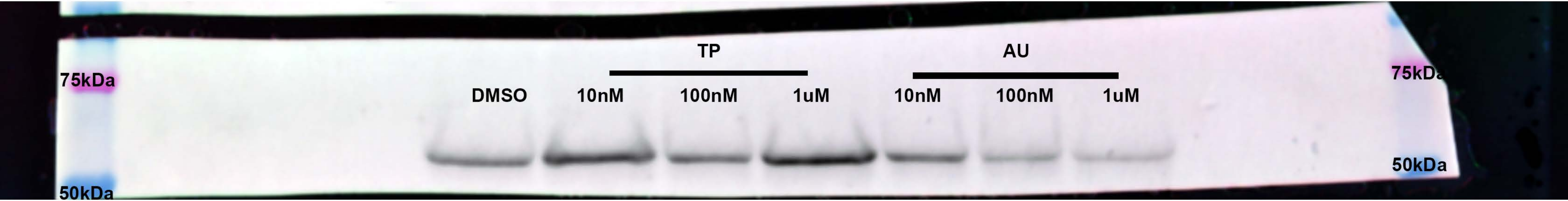

MIA PaCa-2 GemR 24h AKT

Method: Azure
Figure 9B boxed panel)

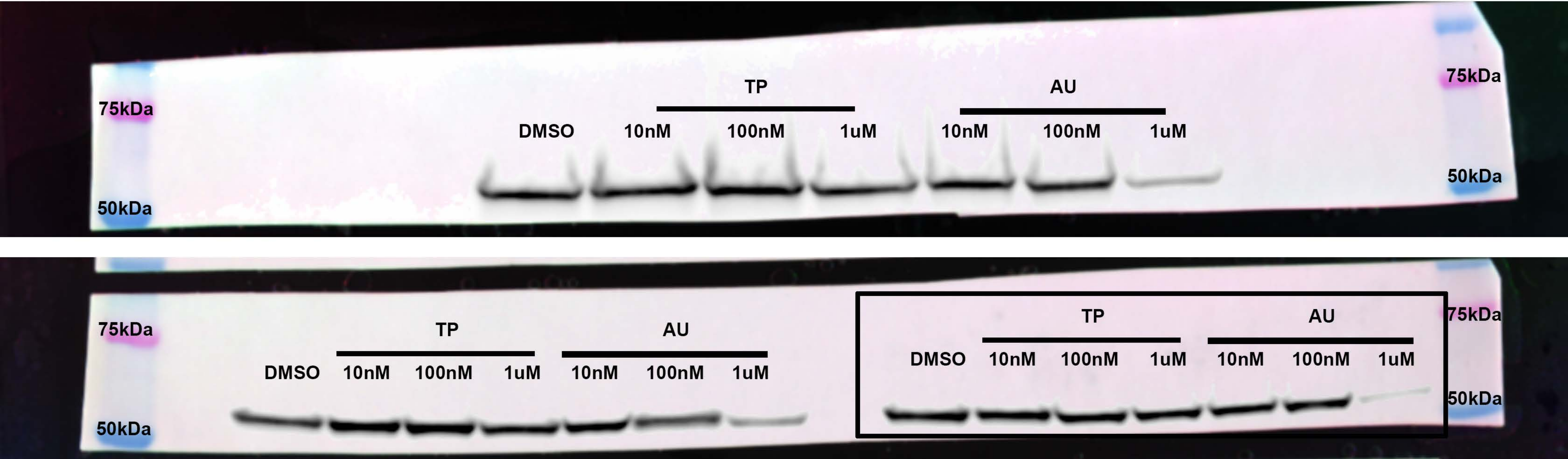

MIA PaCa-2 GemR 24h c-Myc

Method: Azure
Figure 9B boxed panel)

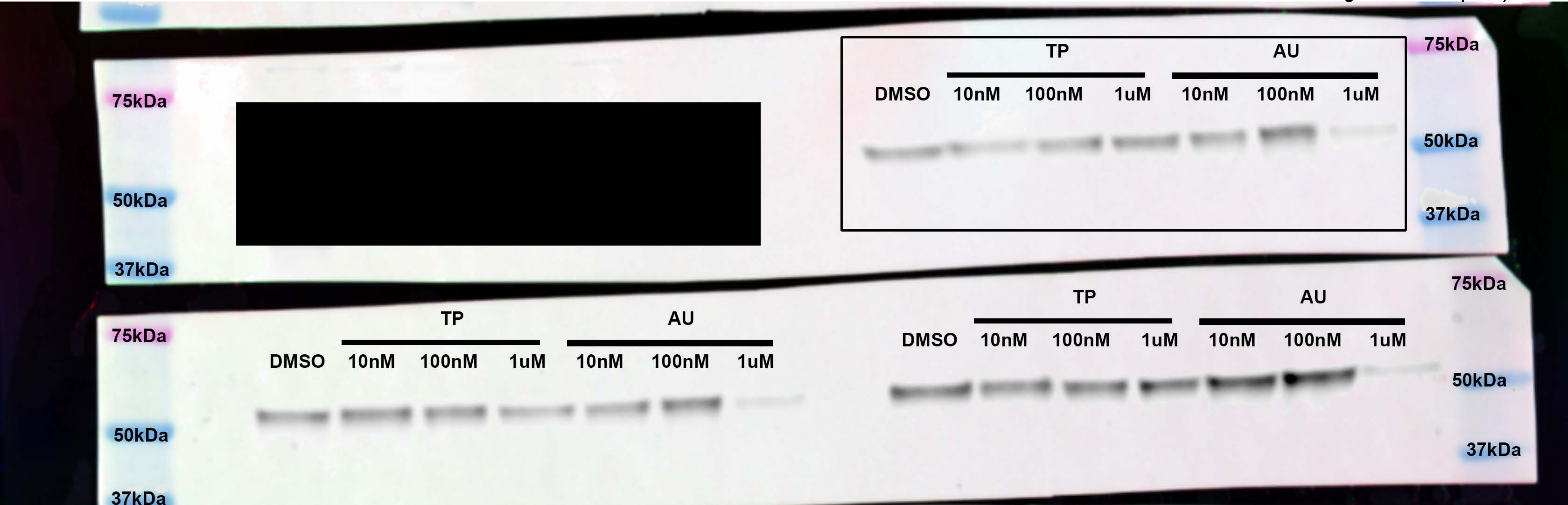

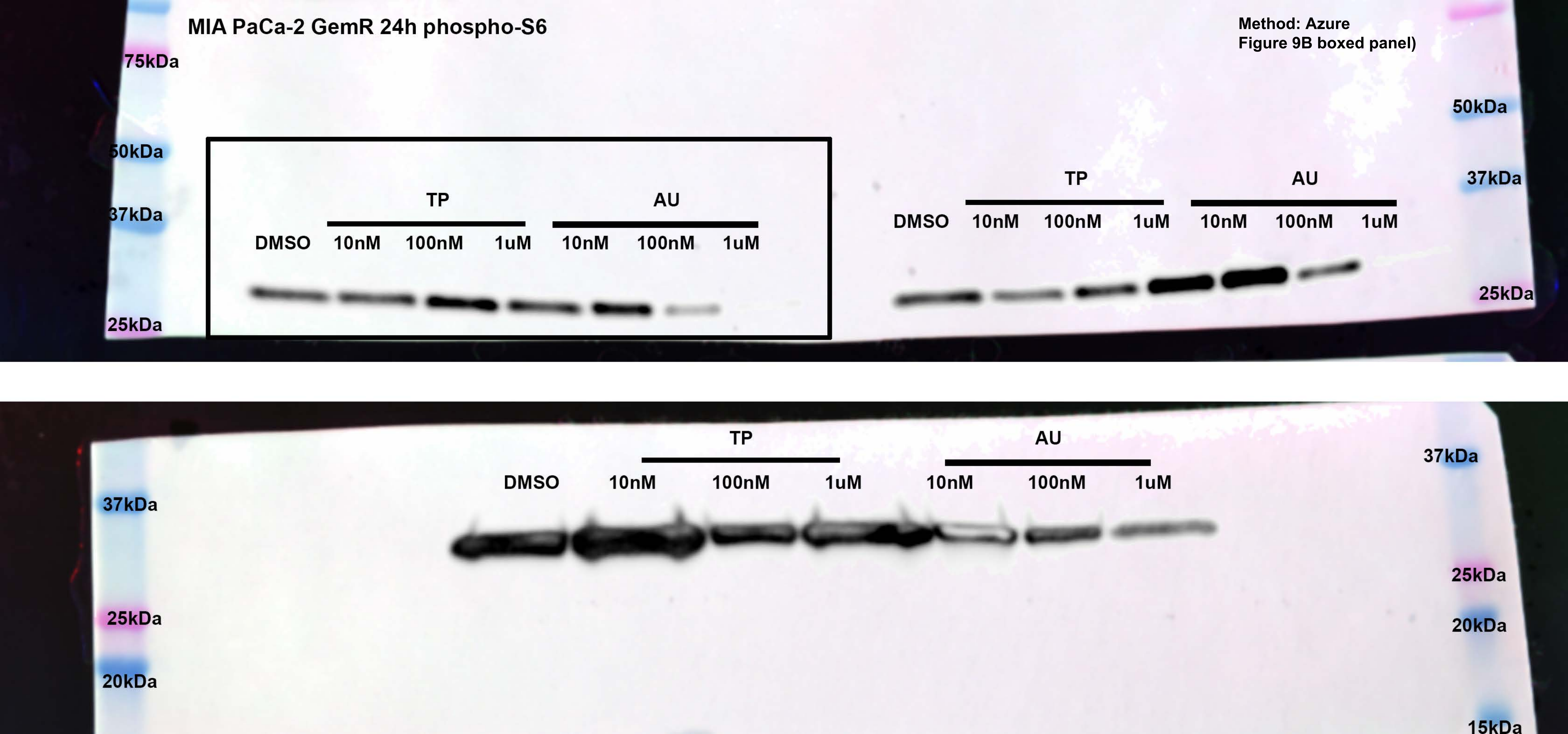
MIA PaCa-2 GemR 24h phospho-S6
Method: Azure
Figure 9B boxed panel)
75kDa
50kDa
37kDa
25kDa
TP
AU
DMSO
10nM
100nM
1uM
10nM
100nM
1uM
50kDa
37kDa
25kDa
TP
AU
DMSO
10nM
100nM
1uM
10nM
100nM
1uM
37kDa
25kDa
20kDa
TP
AU
DMSO
10nM
100nM
1uM
10nM
100nM
1uM
37kDa
25kDa
20kDa
15kDa

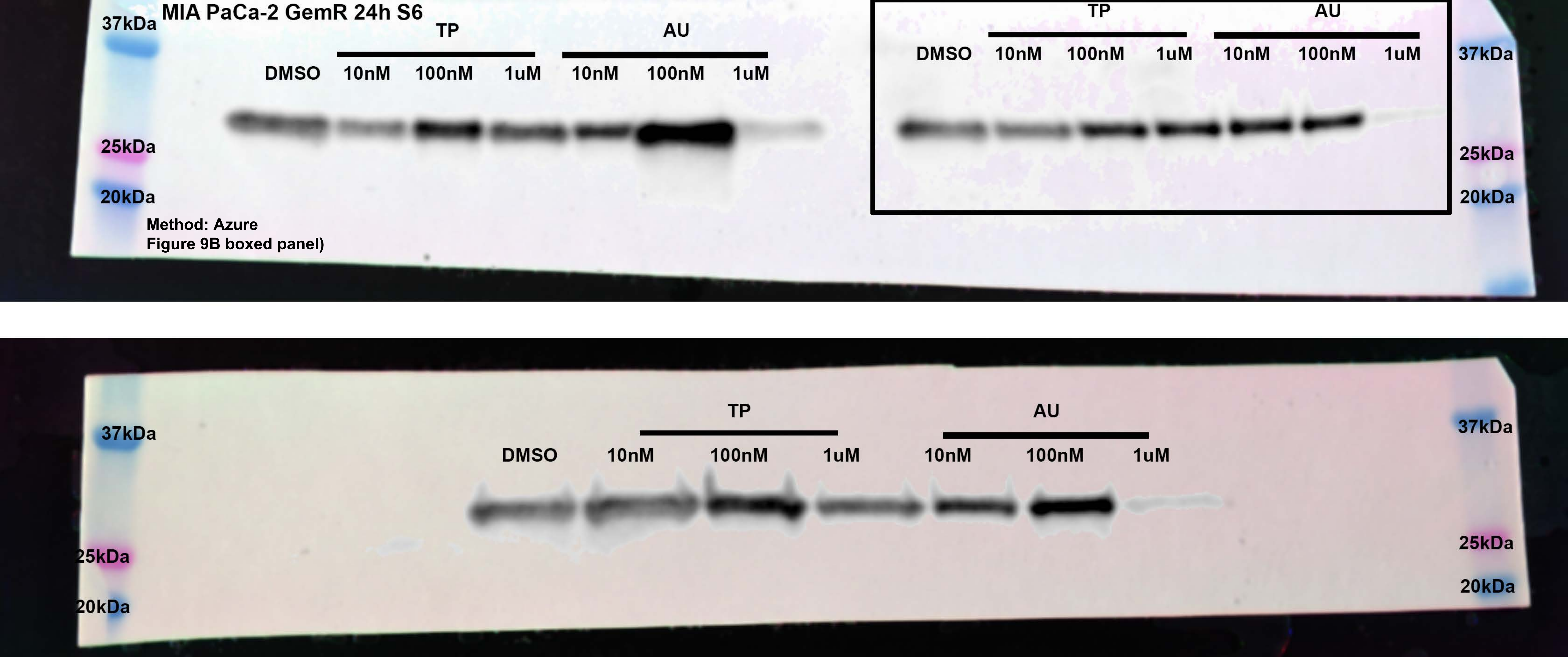
MIA PaCa-2 GemR 24h S6
37kDa
25kDa
20kDa
DMSO
TP
AU
10nM
100nM
1uM
Method: Azure
Figure 9B boxed panel)

MIA PaCa-2 GemR 24h Actin

Method: Azure
Figure 9B boxed panel)

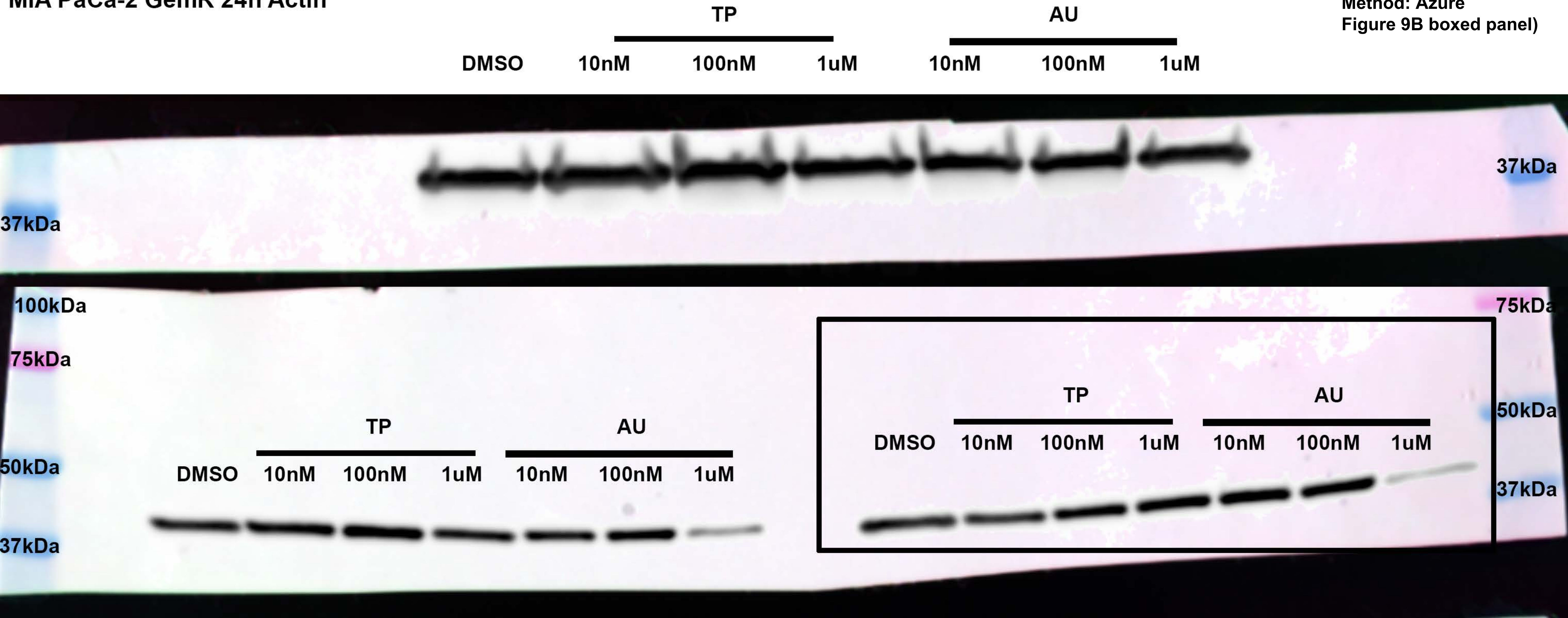

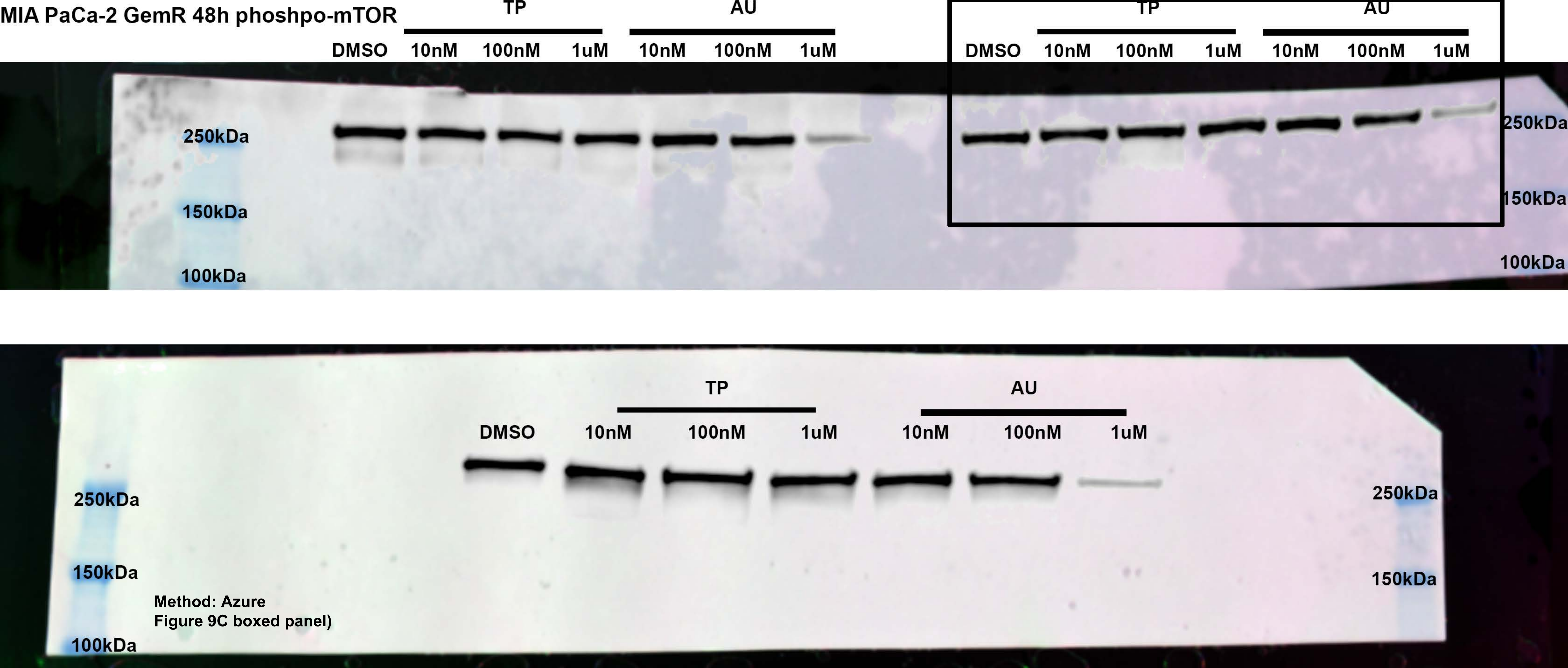
MIA PaCa-2 GemR 48h phoshpo-mTOR
TP
AU
DMSO
10nM
100nM
1uM
10nM
100nM
1uM
250kDa
150kDa
100kDa
Method: Azure
Figure 9C boxed panel)

MIA PaCa-2 GemR 48h mTOR

Method: Azure
Figure 9C boxed panel)

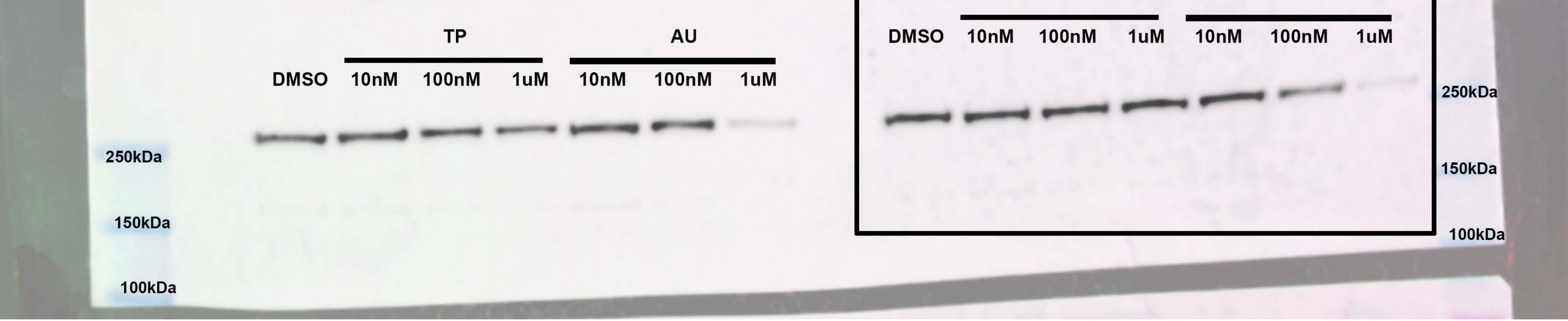

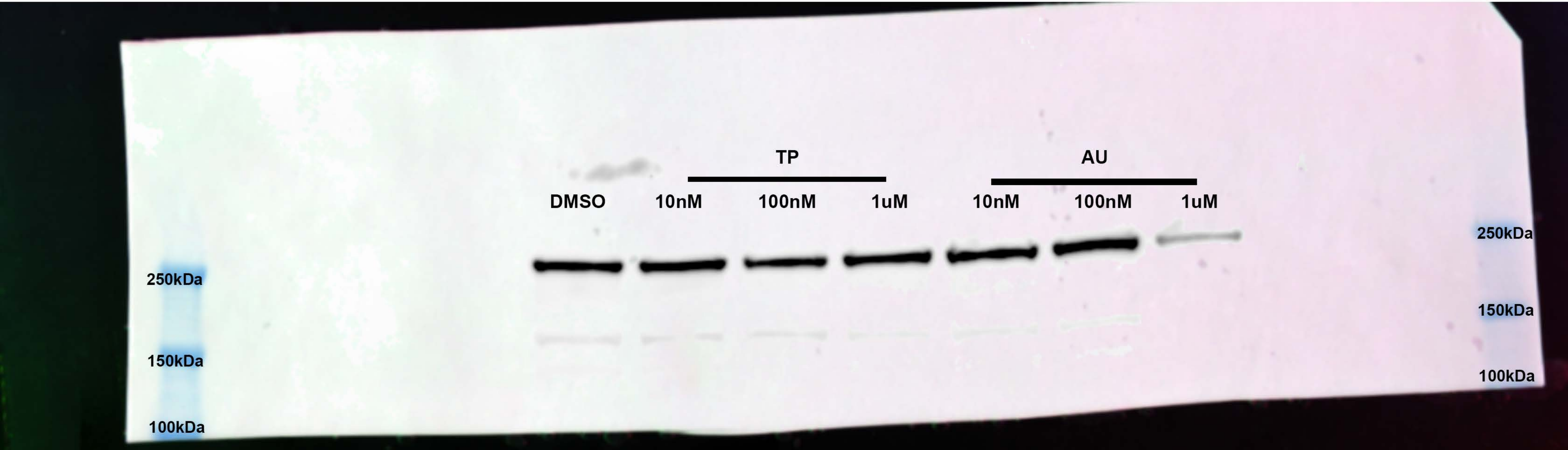

MIA PaCa-2 GemR 48h phospho-AKT

Method: Azure
Figure 9C boxed panel)

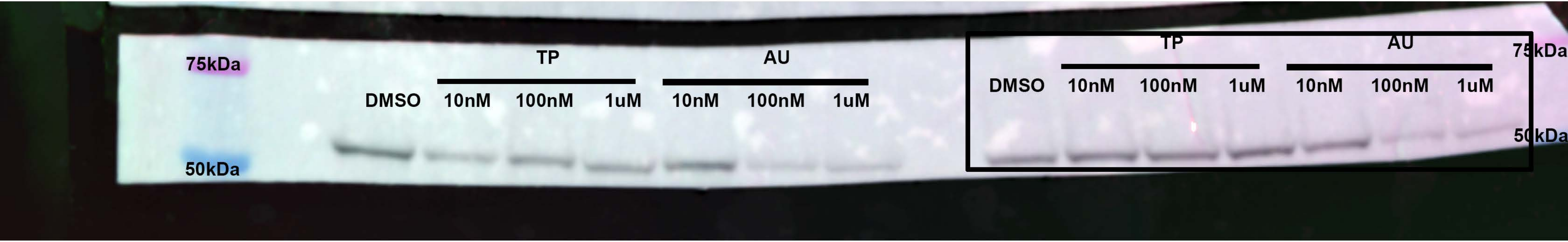

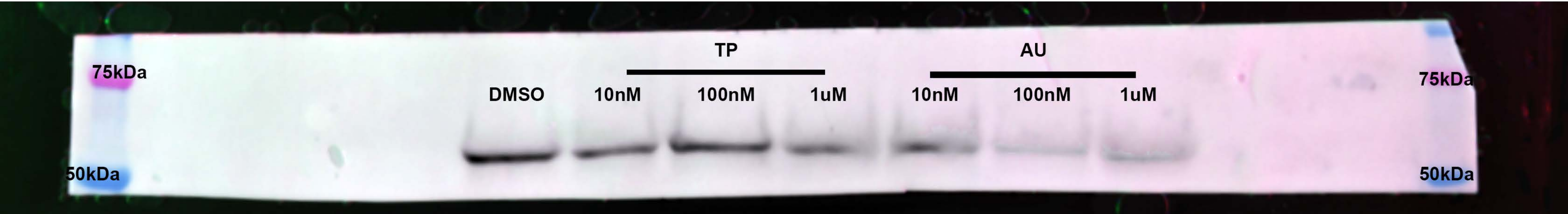

MIA PaCa-2 GemR 48h AKT

Method: Azure
Figure 9C boxed panel)

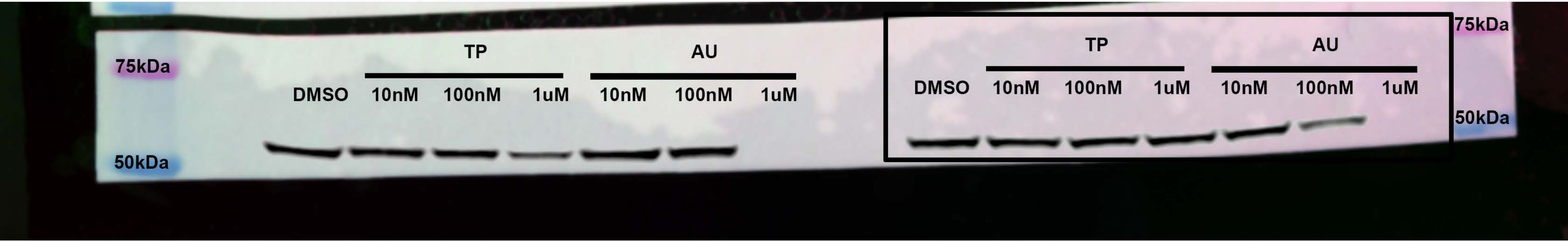

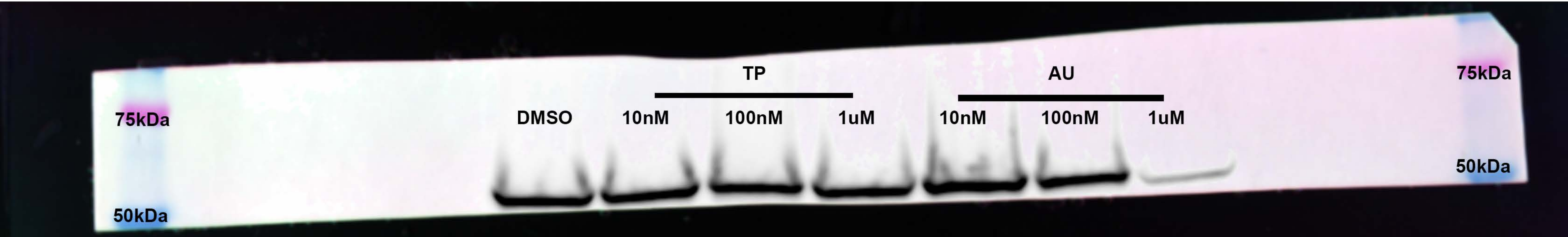

MIA PaCa-2 GemR 48h c-Myc

Method: Azure
Figure 9C boxed panel)

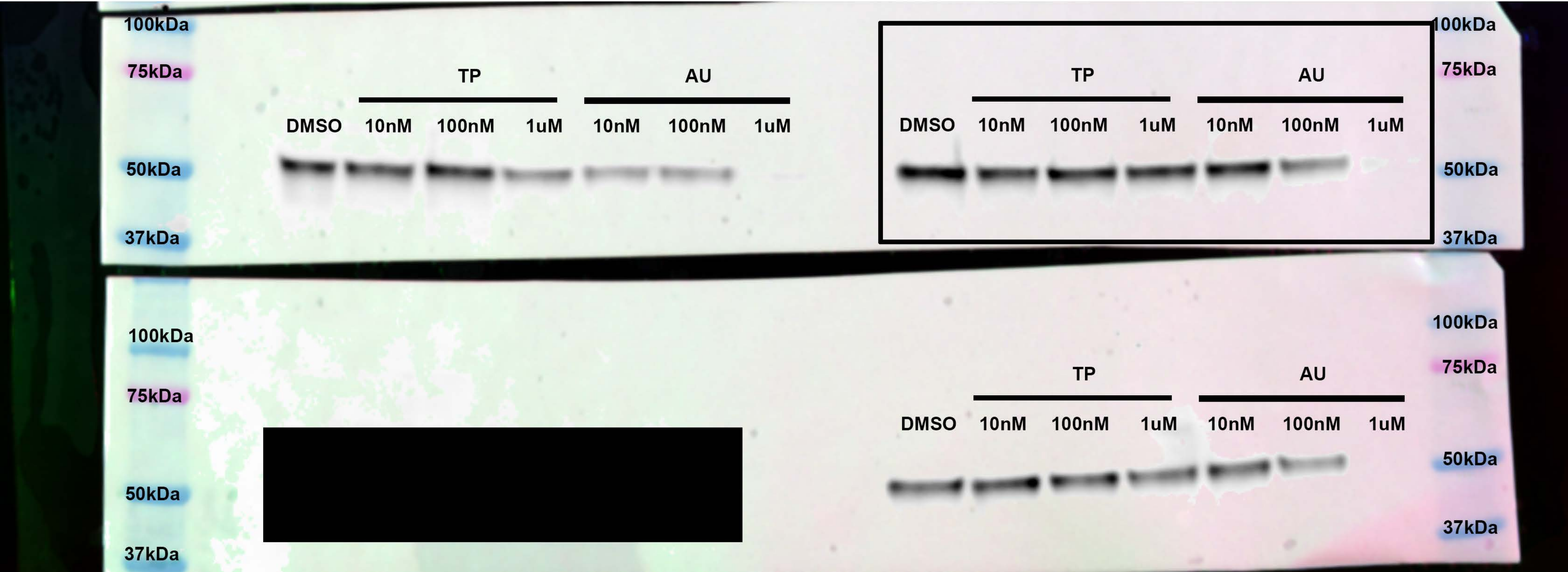

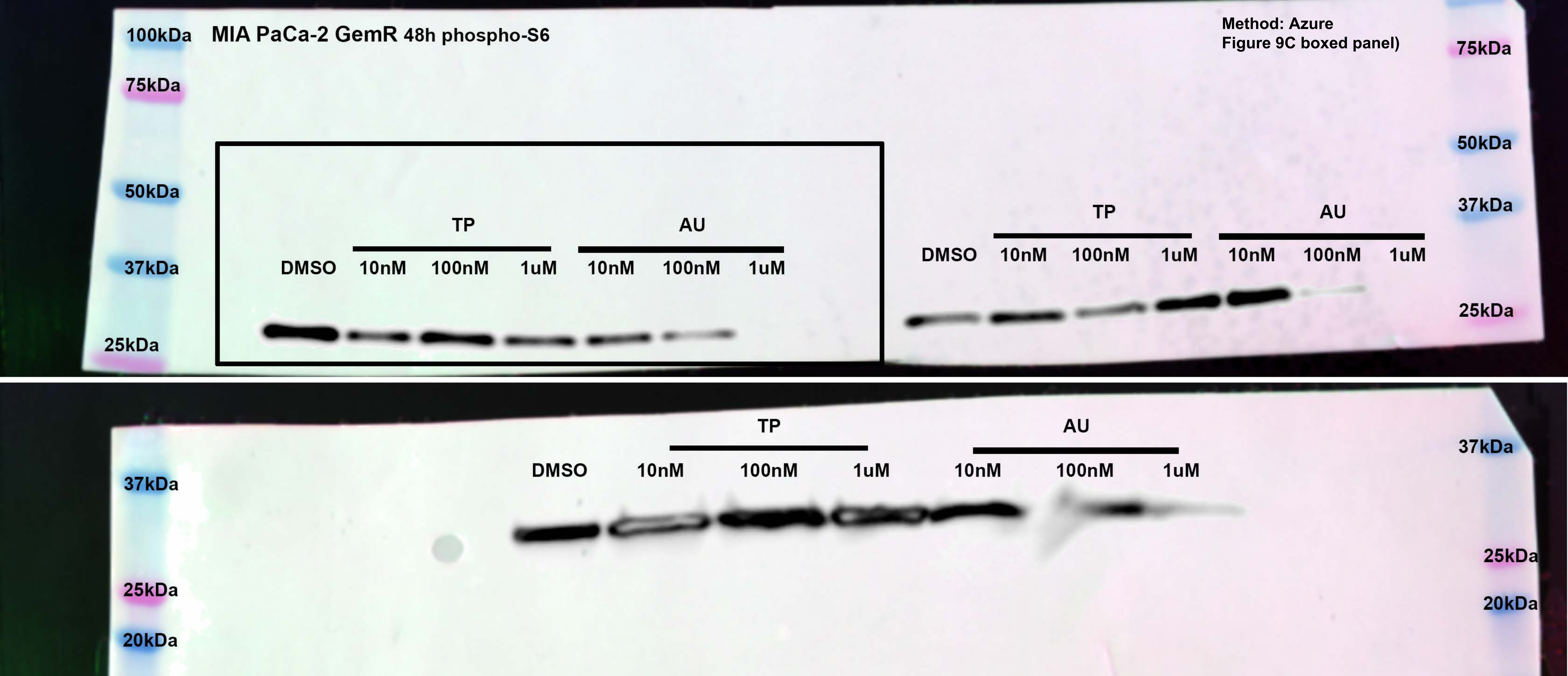

100kDa
MIA PaCa-2 GemR 48h phospho-S6
Method: Azure
Figure 9C boxed panel)
75kDa
75kDa
50kDa
50kDa
37kDa
37kDa
TP
AU
DMSO
10nM
100nM
1uM
10nM
100nM
1uM
TP
AU
DMSO
10nM
100nM
1uM
10nM
100nM
1uM
25kDa
25kDa
TP
AU
DMSO
10nM
100nM
1uM
10nM
100nM
1uM
37kDa
37kDa
25kDa
25kDa
20kDa
20kDa

MIA PaCa-2 GemR 48h S6

Method: Azure
Figure 9C boxed panel)

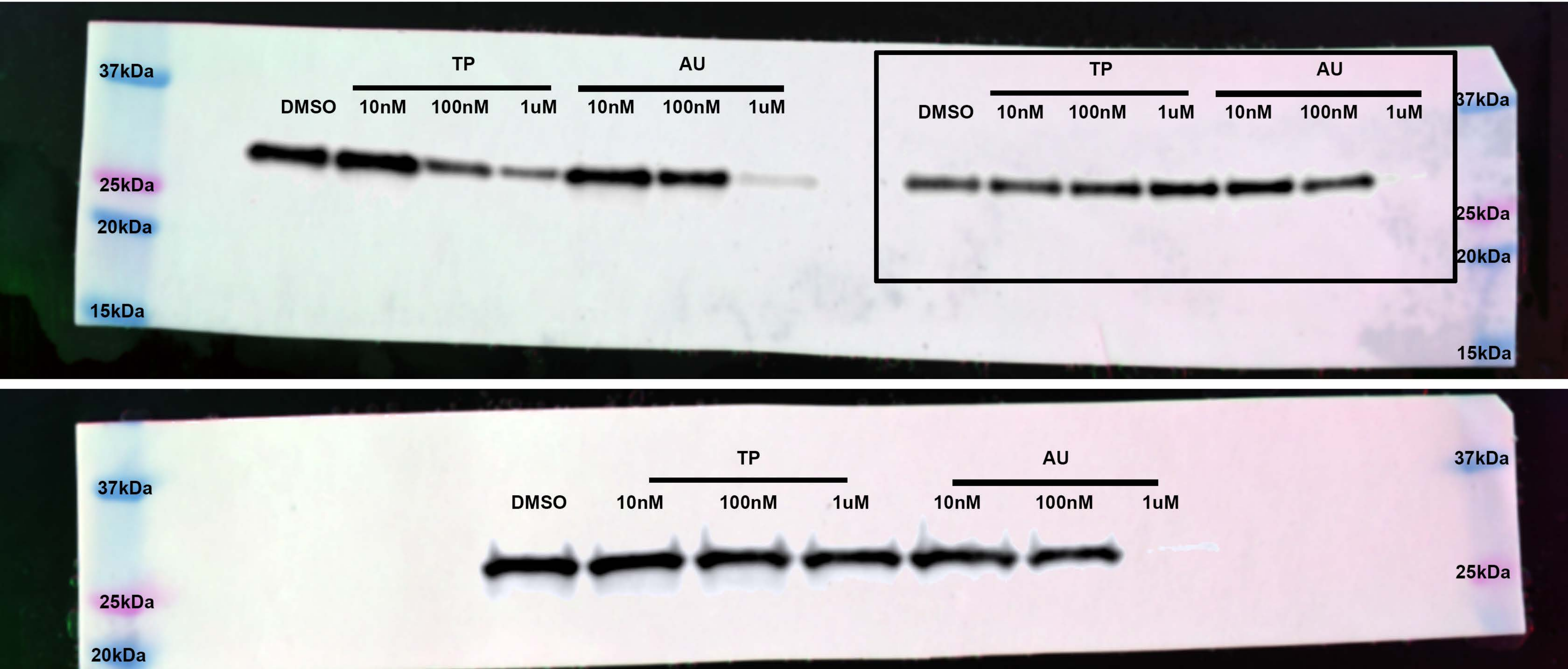

MIA PaCa-2 GemR 48h Actin

Method: Azure
Figure 9C boxed panel)

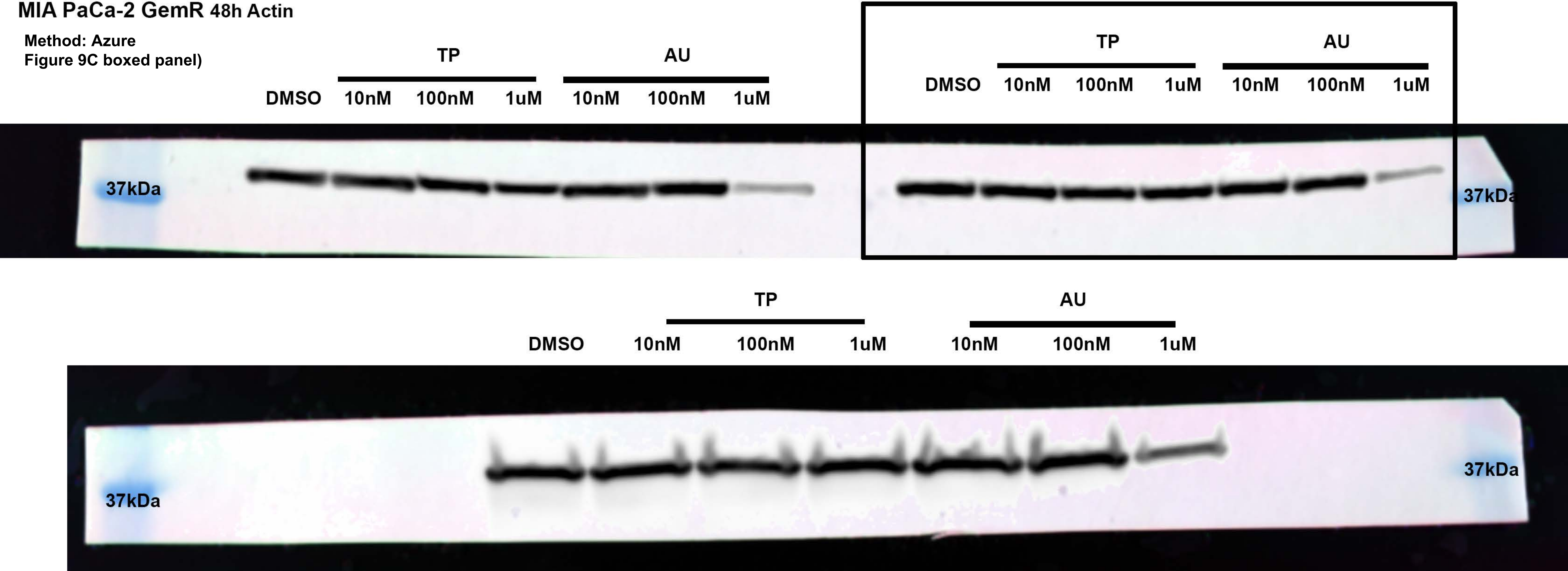

MIA PaCa-2 GemR 72h phospho-mTOR

Method: Azure
Figure 9D boxed panel)

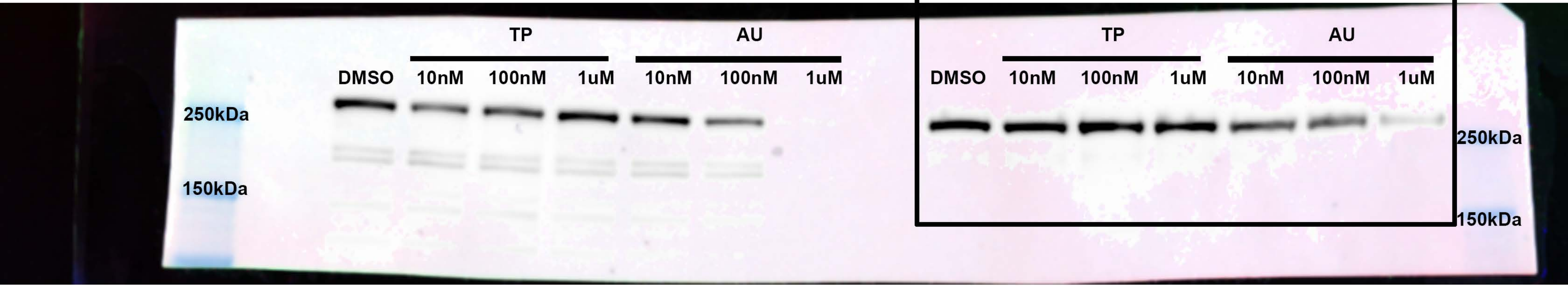

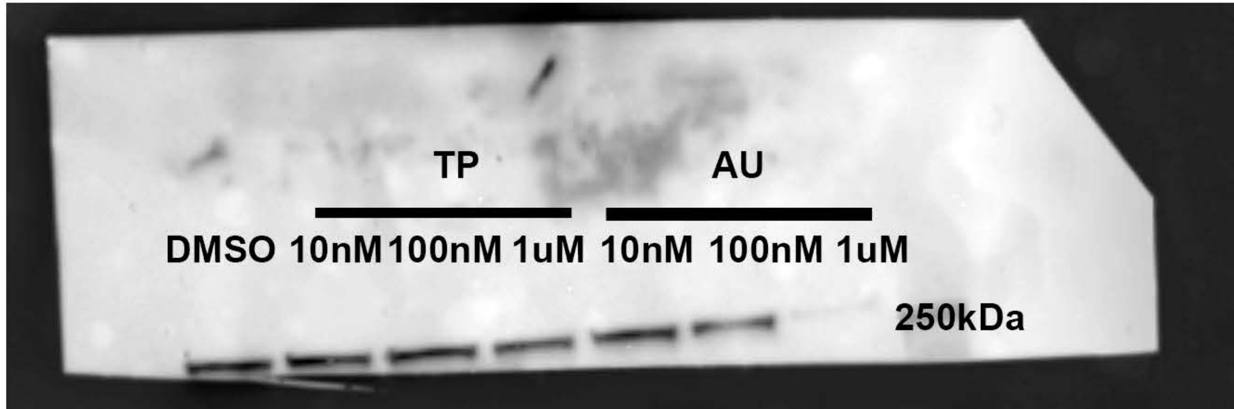

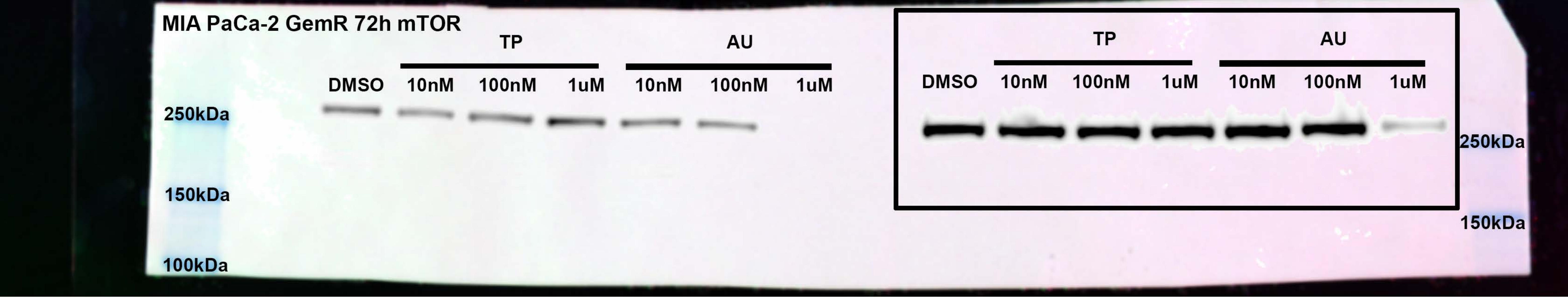

Method: Azure
Figure 9D boxed panel)

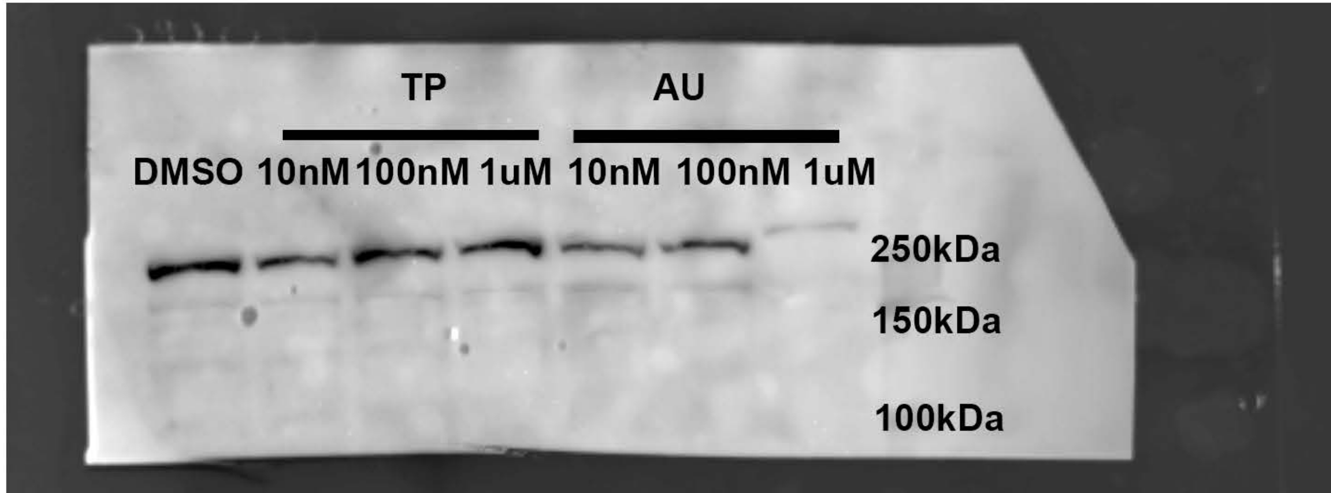

MIA PaCa-2 GemR 72h phospho-AKT

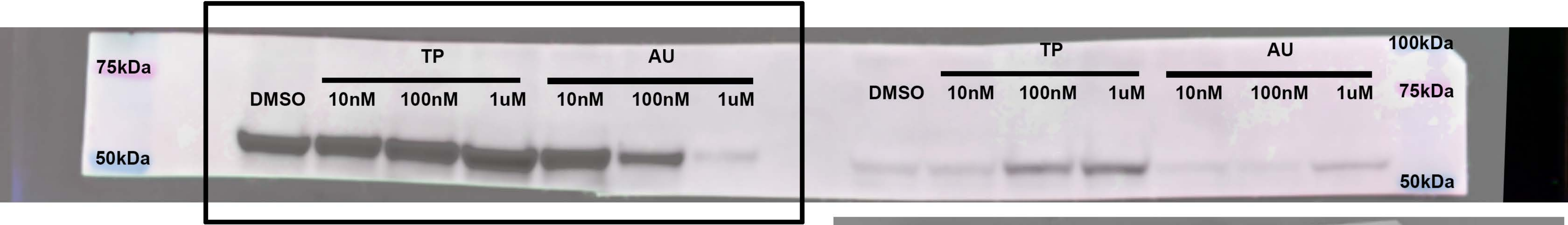

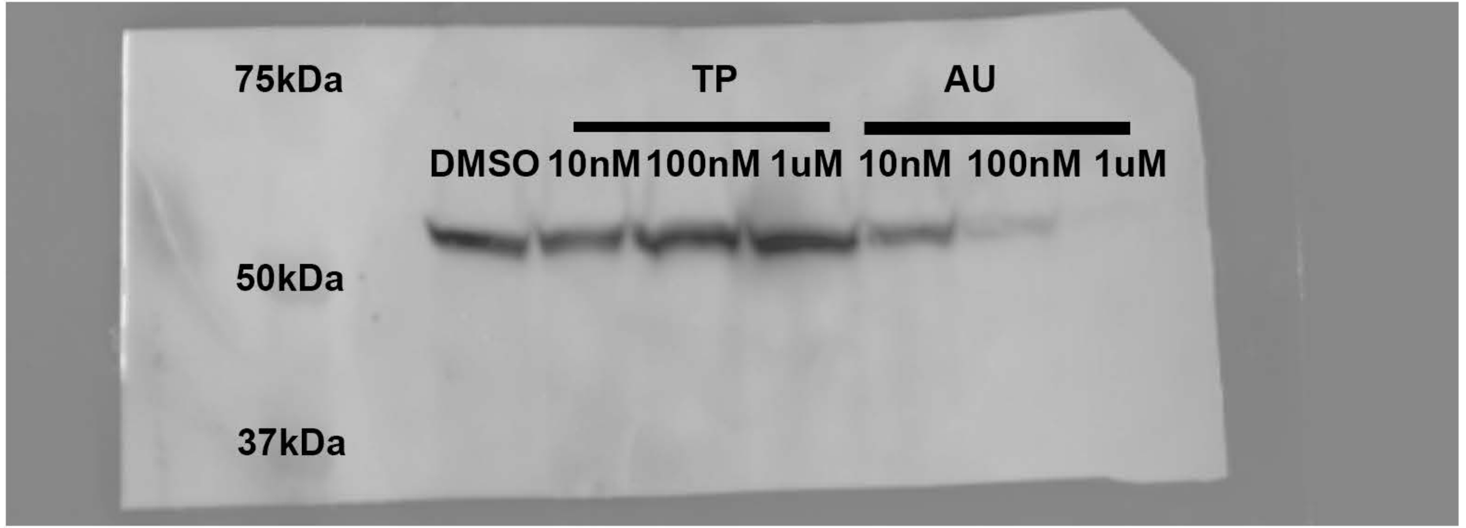

Method: Azure
Figure 9D boxed panel)

MIA PaCa-2 GemR 72h AKT

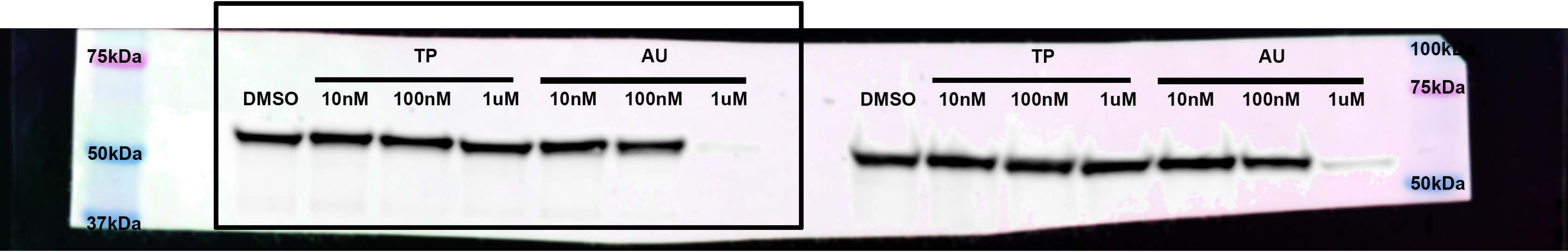

Method: Azure
Figure 9D boxed panel)

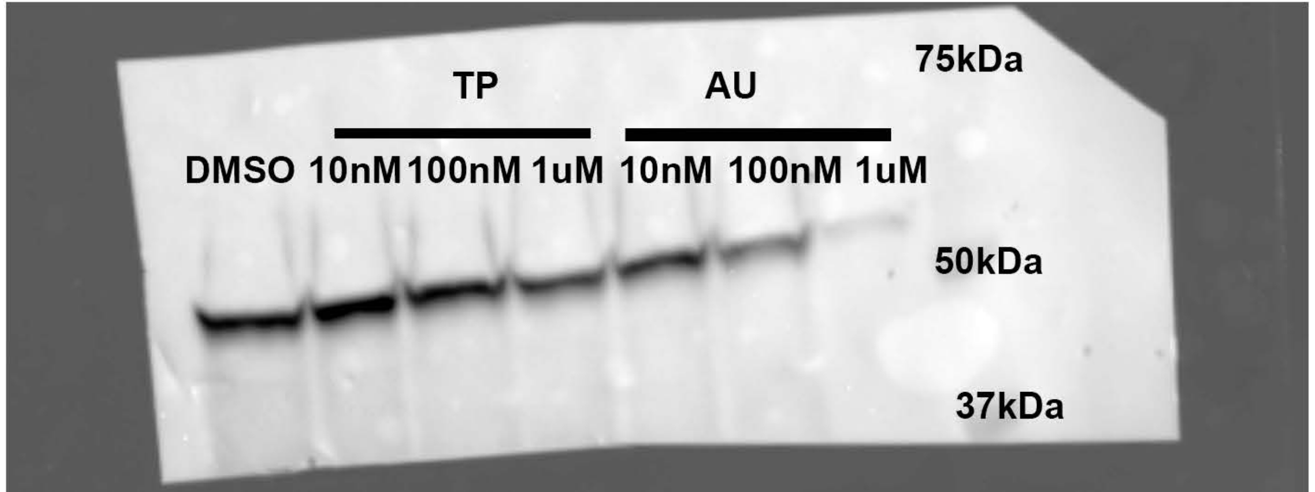

MIA PaCa-2 GemR 72h c-Myc

Method: Azure
Figure 9D boxed panel)

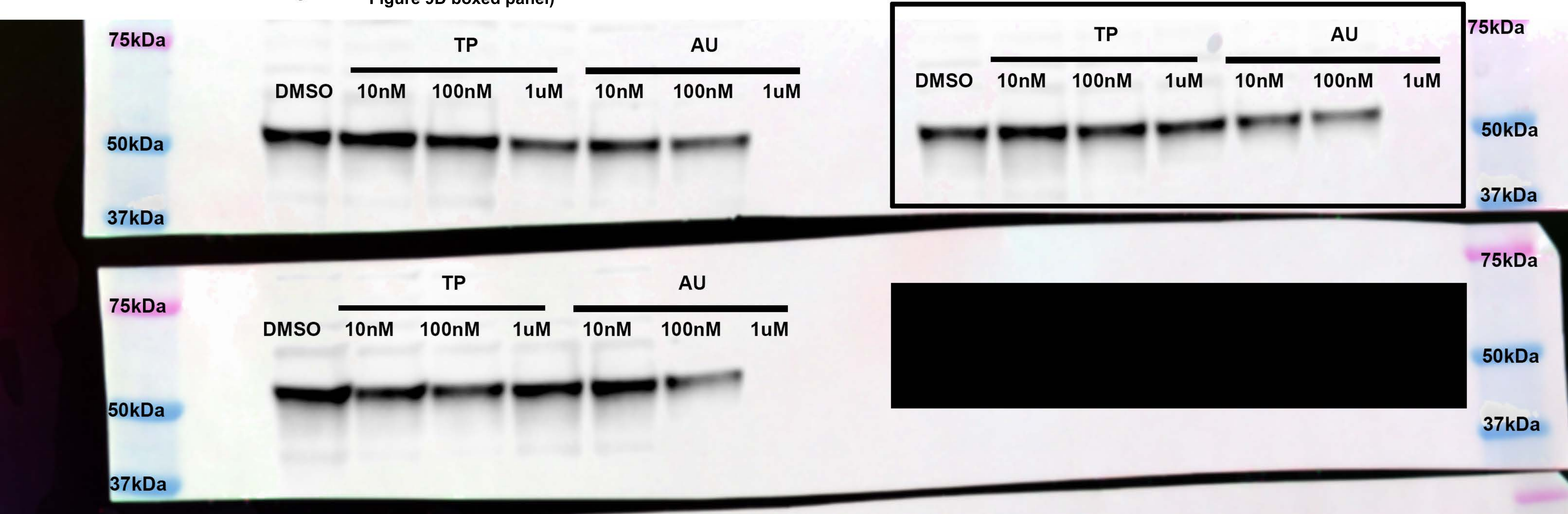

MIA PaCa-2 GemR 72h phospho-S6

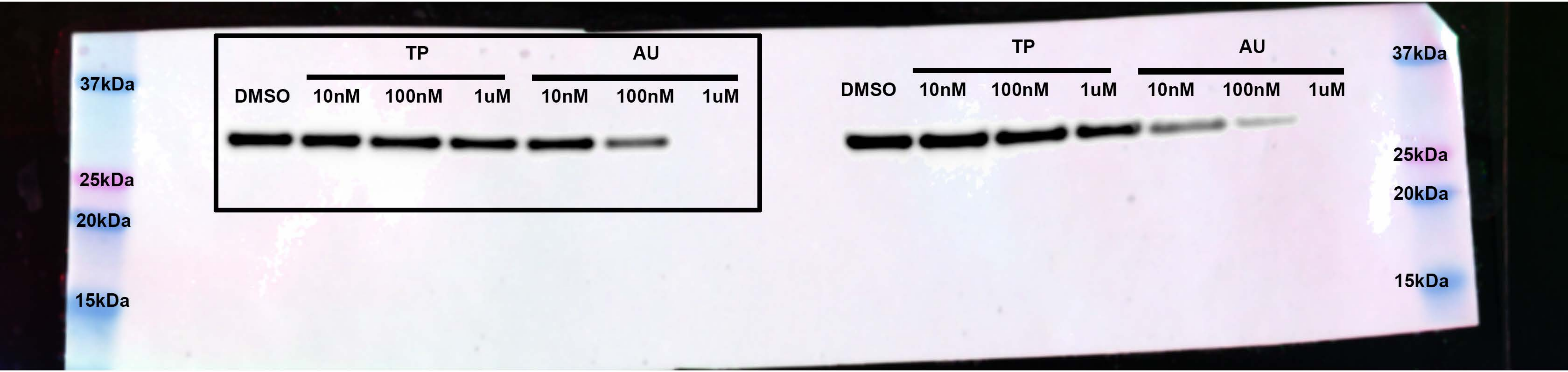

Method: Azure
Figure 9D boxed panel)

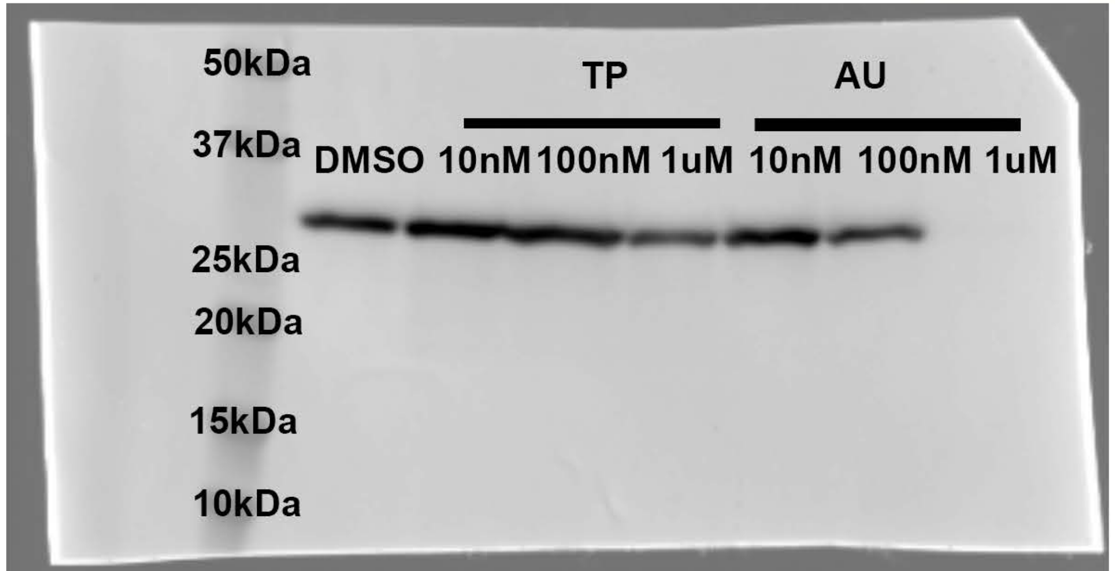

MIA PaCa-2 GemR 72h S6

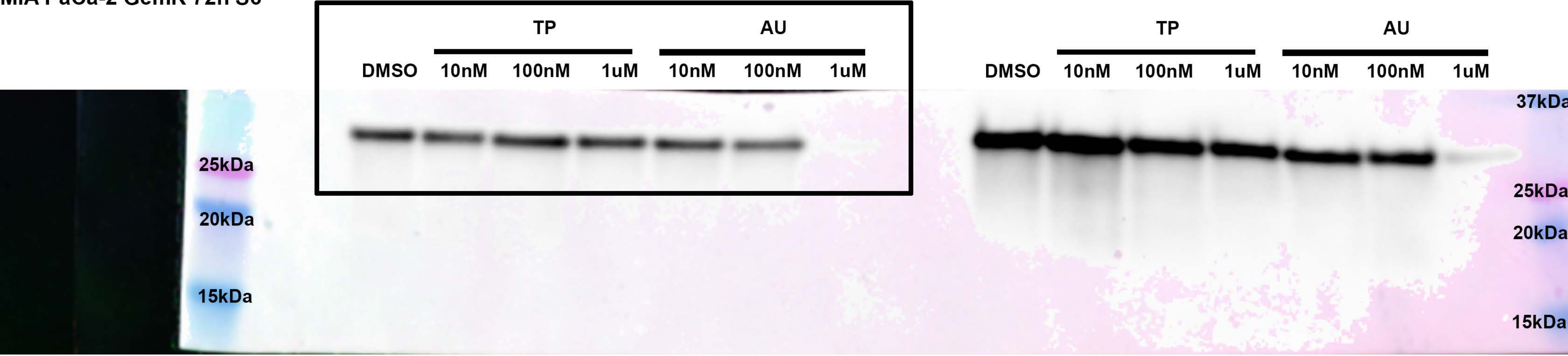

Method: Azure
Figure 9D boxed panel)

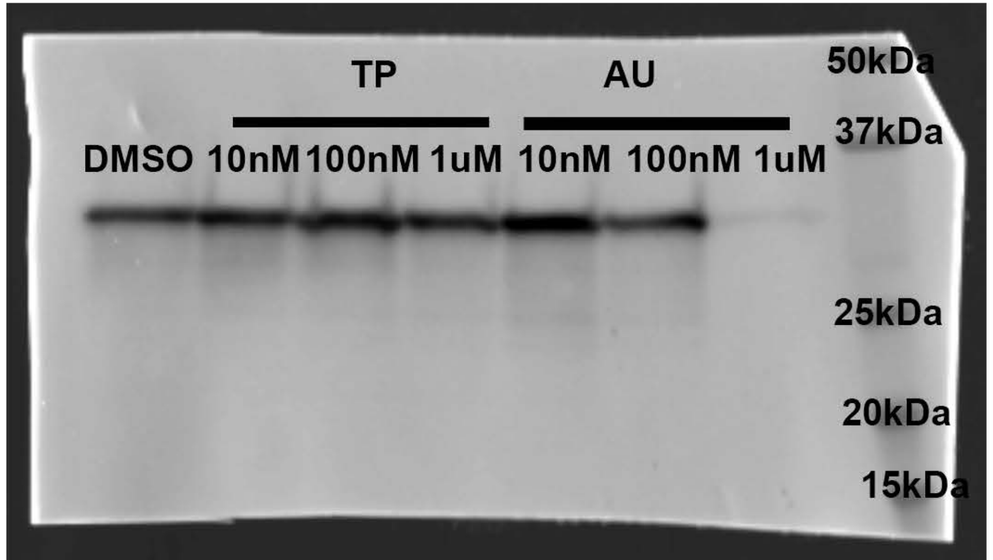

MIA PaCa-2 GemR 72h Actin

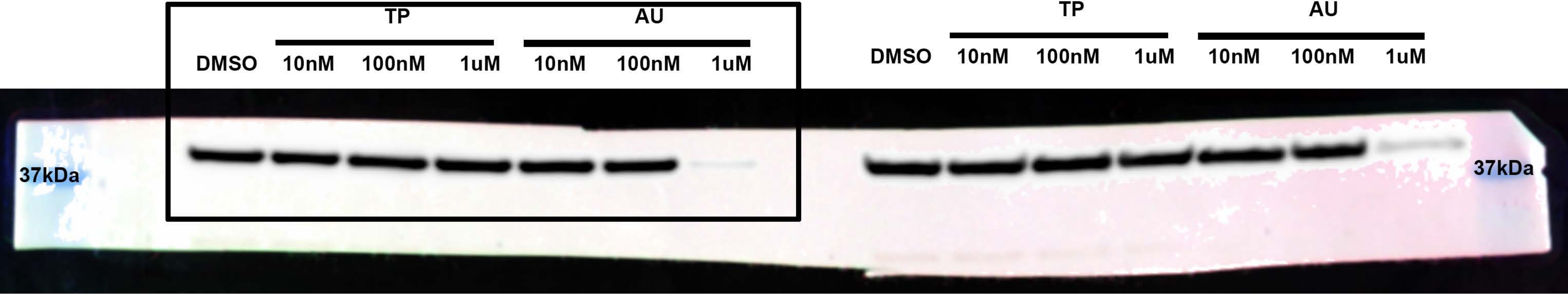

Method: Azure
Figure 9D boxed panel)

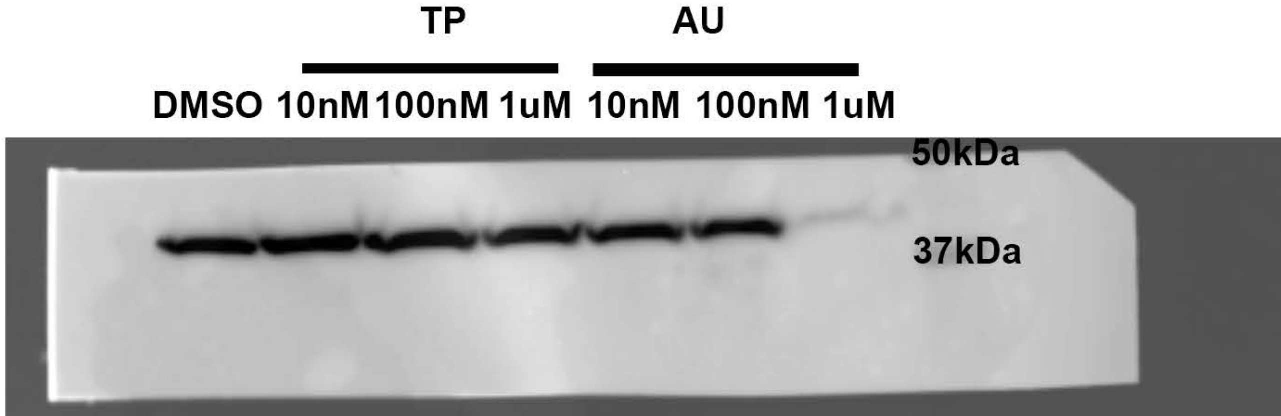

Supplement: S1 Raw images — (PDF) [file pone.0294065.s004.pdf]
