# Supplementary material for: AUM302, a novel triple kinase PIM/PI3K/mTOR inhibitor, is a potent in vitro pancreatic cancer growth inhibitor
Source: PLoS One. 2023 Nov 9;18(11):e0294065. doi: 10.1371/journal.pone.0294065 (PMC10635512; doi:10.1371/journal.pone.0294065)
Supplement: Attachment — Submitted filename: Response to the Reviewers comments.pdf [file pone.0294065.s005.pdf]

Renaissance School of Medicine
Stony Brook University

Agnieszka B. Bialkowska, PhD
Associate Professor, Department of Medicine
Renaissance School of Medicine at Stony Brook University

September 12th, 2023

Editor and Reviewers *PLOS ONE*
Re: PONE-D-23-13615
AUM302, a novel triple kinase PIM/PI3K/mTOR inhibitor, is a potent pancreatic cancer growth inhibitor

Dear Editor and Reviewers,

We want to thank the Reviewers for their invaluable comments. We also acknowledge the concerns raised by the Reviewers and now provide a revised copy of the manuscript, considering all of the Reviewers' concerns. This revised manuscript addressed all the comments and suggestions the Editor and Reviewers provided. Please see our responses to your comments below.

Reviewer 1.

Comment 1. The authors should also include a PI3K/mTOR inhibitor to compare with and really demonstrate that the triple inhibitor is more potent.

Response 1. We agree with the Reviewer comment. We performed cell viability studies in BxPC-3, Capan-2, MIA PaCa-2, PANC-1, and Hs766T using two compounds, GDC-0941 and BEZ235, both PI3K and mTOR inhibitors. We included the results in Figure 1 and Table 1 and added an appropriate statement in the Results section.

Comment 2. Correct table 1. When a cell line is resistant to a drug de GI50 should be higher than the highest concentration tested.

Response 2. We provided IC50 values in nanomolar concentrations. We performed all experiments using low concentrations of AUM-302 of 10nM and 100nM as we have observed its significant effects using these concentrations on cell proliferation and cell cycle of tested PDAC cell lines.

Comment 3. In order to be able to take any conclusions regarding the effect in biomarker modulation, on one hand the authors should provide a quantification of the bands and in the case of phosphorylation take into account the total levels of each corresponding protein. Moreover, 72h is a very long time treatment to see effect in phosphorylation as compensatory mechanisms could take place.

Response 3. We agree with the Reviewer's comment. In this revised manuscript, we included the results of the 24-hour treatment of five tested PDAC cell lines with 10nM and 100nM concentrations of TP-3654 and AUM-302 in Figure 6 and the quantification of the western blot results in Figure 7.

Comment 4. In the case of resistant cell line, the highest concentration tested of AUM-302 for the evaluation of biomarkers has a very strong effect in proliferation at 72h then this results are not reliable. Shorter treatments should be included.

Response 4. We provided western blot results from 24-, 48- and 72-hour treatment in Figure 9 and appropriate quantifications of the western blot results in Supplementary Figures 1 through 3.

Stony Brook University Medical Center | Department of Medicine
Health Sciences Center T-17, Room 90, 101 Nicolls Road, Stony Brook, NY 11794-8176
Tel: (631) 638-2161; Fax: (631) 865-5882; Email: Agnieszka.Bialkowska@stonybrookmedicine.edu

Agnieszka B. Bialkowska, PhD
Associate Professor, Department of Medicine
Renaissance School of Medicine at Stony Brook University

Reviewer 2.

Comment 1. Why authors did not determine PIM1/2 inhibition to ensure the correlation with the demonstrated results and the correlation of inhibited molecular components of PIM/PI3K/m TOR?

Response 1. The AUM-302 compound does not impact the levels of PIM1/2 on RNA or protein levels. The compound has been designed to affect their activity. Thus, the levels of PIM1/2 are unchanged, but their activities are decreased upon AUM-302 treatment.

Comment 2. It is advisable for author to make a semiquantitative analysis of western plot bands to facilitate the interpretation of inhibited proteins.

Response 2. We agree with the Reviewer's suggestions. We performed appropriate quantifications and included them in Figure 7 and Supplementary Figures 1 through 3.

Comment 3. Table one should include standard deviation of demonstrated IC50s, its title should be also more elucidated to be self-explanatory.

Response 3. Thank you for your suggestion. We modified the title of Table 1 and provided IC50 values in nanomolar concentrations with standard deviations.

Comment 4. Quality of Figures 2-8 should be improved in their quality.

Response 4. All figures were prepared in Adobe Photoshop according to the PLOS ONE requirements with at least 300-600 dpi. All figures are submitted as .tif files. In addition, we utilized Preflight Analysis and Conversion Engine (PACE) digital diagnostic tool recommended by PLOS ONE that tests the quality of prepared images and their compliance with the journal requirements. It could be that the journal provides low-quality figures for the primary review. We hope that the quality of the revised figures is adequate.

Comment 5. Authors should reconstruct the discussion part to be more focused on discussing the demonstrated results and the correlation of inhibited molecular components of PIM/PI3K/mTOR.

Response 5. We want to thank the Reviewer for this suggestion. We revised the Discussion section and added more information relevant to inhibiting PIM/PI3K/mTOR pathway components.

Reviewer 3.

Comment 1. The authors should add the words in vitro to this title.

Response 1. We modified the title of the manuscript to reflect its in vitro studies.

Comment 2. Since the authors did not carry out in vivo studies with either intra-pancreatic or autochthonous models of PDAC, the authors should add a statement or two to the discussion section to indicate that such studies are necessary to determine whether AUM302 can be effective in vivo, and whether AUM302 is capable of altering in a beneficial manner the immune-excluding tumor microenvironment in this immune-cold cancer.

Agnieszka B. Bialkowska, PhD
Associate Professor, Department of Medicine
Renaissance School of Medicine at Stony Brook University

Response 2. Thank you for this suggestion. We modified the Discussion section and added a statement emphasizing the necessity for in vivo studies to assess AUM-302 full impact on the PDAC microenvironment.

We confirm that neither the manuscript nor any parts of its content are currently under consideration or published in another journal. We changed the revised manuscript to ascertain compliance with the PLOS ONE journal's requirements.

We hope the incorporated changes will satisfy the Reviewers and Editor and render the revised manuscript suitable for publication. Thank you for being so considerate.

All authors have approved the manuscript and agree with its submission to *PLOS ONE.*

Please feel free to contact me if I can be of further assistance,

Sincerely Yours,

Agnieszka Bialkowska

Agnieszka B. Bialkowska, PhD
Associate Professor
Renaissance School of Medicine at Stony Brook University
Department of Medicine
GI Translational Research Lab
HSC-T17 Room 090
Stony Brook, NY 11794-8176
Phone: (631) 638 2161
Email: Agnieszka.Bialkowska@stonybrookmedicine.edu
